# Adipose-Derived Stem Cells Facilitate Ovarian Tumor Growth and Metastasis by Promoting Epithelial to Mesenchymal Transition Through Activating the TGF-β Pathway

**DOI:** 10.3389/fonc.2021.756011

**Published:** 2021-12-22

**Authors:** Xiaowu Liu, Guannan Zhao, Xueyun Huo, Yaohong Wang, Gabor Tigyi, Bing-Mei Zhu, Junming Yue, Wenjing Zhang

**Affiliations:** ^1^ Department of Urology, The First Affiliated Hospital of China Medical University, Shenyang, China; ^2^ Department of Pathology and Laboratory Medicine, The University of Tennessee Health Science Center, Memphis, TN, United States; ^3^ Center for Cancer Research, College of Medicine, The University of Tennessee Health Science Center, Memphis, TN, United States; ^4^ School of Basic Medical Sciences, Capital Medical University, Beijing, China; ^5^ Department of Pathology, Immunology, and Microbiology, Vanderbilt University Medical Center, Nashville, TN, United States; ^6^ Department of Physiology, College of Medicine, The University of Tennessee Health Science Center, Memphis, TN, United States; ^7^ Regenerative Medicine Research Center, West China Hospital, Sichuan University, Chengdu, China; ^8^ Department of Genetics, Genomics & Informatics, College of Medicine, The University of Tennessee Health Science Center, Memphis, TN, United States

**Keywords:** adipose-deprived stem cell, ovarian cancer, metastasis, epithelial to mesenchymal transition (EMT), TGF-β

## Abstract

Adipose-derived stem cells (ADSC) are multipotent mesenchymal stem cells derived from adipose tissues and are capable of differentiating into multiple cell types in the tumor microenvironment (TME). The roles of ADSC in ovarian cancer (OC) metastasis are still not well defined. To understand whether ADSC contributes to ovarian tumor metastasis, we examined epithelial to mesenchymal transition (EMT) markers in OC cells following the treatment of the ADSC-conditioned medium (ADSC-CM). ADSC-CM promotes EMT in OC cells. Functionally, ADSC-CM promotes OC cell proliferation, survival, migration, and invasion. We further demonstrated that ADSC-CM induced EMT *via* TGF-β growth factor secretion from ADSC and the ensuing activation of the TGF-β pathway. ADSC-CM-induced EMT in OC cells was reversible by the TGF-β inhibitor SB431542 treatment. Using an orthotopic OC mouse model, we also provide the experimental evidence that ADSC contributes to ovarian tumor growth and metastasis by promoting EMT through activating the TGF-β pathway. Taken together, our data indicate that targeting ADSC using the TGF-β inhibitor has the therapeutic potential in blocking the EMT and OC metastasis.

## Introduction

Ovarian cancer (OC) is the most lethal gynecological malignancy worldwide, because of its early dissemination in the peritoneal cavity, late detection, and high recurrence rate (1). Most patients are diagnosed at advanced stages with poor prognosis and significant mortality. OC metastasizes *via* pelvic dissemination directly from the primary tumor to peritoneal organs, which is usually asymptomatic at the early stage (2).

The tumor microenvironment (TME) plays a critical role in tumor progression and metastasis, which is becoming a potential therapeutic target (3). TME is composed of fibroblast, mesenchymal stromal cells (MSC), immune cells, blood vessels, and extracellular matrix. MSC has been shown to play different roles in different cancers, including colon, breast, pancreatic, and lung cancer. The influences of MSC on cancers are controversial. MSC in the TME have been shown to promote the aggressive malignant phenotypes of various solid cancers, including colon cancer (4) and breast cancer (5). However, MSC also showed inhibitory effects in lung cancer (6) and liver cancer (7).

Adipose-derived stem cells (ADSC) are residents in adipose tissue and share most multipotency features of MSC. ADSC can differentiate into adipocyte, osteoblast, chondrocyte, myocyte, and lineages. Several studies have demonstrated the interplay between ADSC and cancer cells (8). Intravenous-injected ADSC can promote breast cancer growth and metastasis in a mouse model (9). The effects of ADSC in promoting tumor metastasis have also been observed in pancreatic, prostate, endometrial, and lung cancers (10–14). Human ADSC promotes invasion of breast cancer cells by producing CCL5 *in vitro* (15). ADSC also shows enhanced adipogenic differentiation in breast cancer (16). ADSC increase OC cell proliferation, migration, and chemoresistance (17–19). The conditioned medium (CM) of ADSC alters the proteomic profile of OC cell lines *in vitro* and expression of thymosin beta 4 X-linked (TMSB4X) in OC cells increased significantly, which promoted OC progression (20). Inhibition of TMSB4X attenuated the protumor effects of ADSCs (20). ADSC were reported to promote autophagy through activating the STAT3 signaling pathway (21). ADSC promote cancer progression by differentiating into cancer-associate fibroblast (CAF) or cancer-associate adipocytes (CAA) (22, 23) and facilitating CSC self-renewal (24). In addition, ADSC promote OC chemoresistance *via* inhibiting the cleavage of caspase-3 and inducing the platinum accumulation in OC cells (25, 26). Although, most studies showed protumor effects of ADSC on OC. The inhibitory effects of ADSC on OC progression was also reported, co-culturing ADSC with OC cells inhibited OC cell invasion induced apoptosis (27). ADSC-CM blocked cell cycle and induced apoptosis in OC cell lines (28).

Epithelial to mesenchymal transition (EMT) contributes to malignant tumor progression. ADSC promotes EMT in different tumors, including lung cancer, breast cancer, and glioma (29–31). In the present study, we assessed the effects of ADSC on OC metastasis using CM from ADSC *in vitro* and in an orthotopic OC mouse model *in vivo*. Here, we demonstrate that ADSC contributes to OC metastasis by promoting EMT at least in part *via* activating the TGF-β pathway.

## Material and Methods

### Cell Culture and Preparation of CM

Ovarian cancer cell lines OVCAR3 and OVCAR8 were purchased from NCI and cultured in 10% RPMI1640 medium with 10% FBS (Hyclone, Logan, UT, USA), 100 U/ml penicillin, and 100 μg/ml streptomycin (Invitrogen, Carlsbad, CA, USA). We selected OVCAR3 and OVCAR8 based on their properties inducing OC tumor metastasis and p53 mutational status. Both OVCAR3 and OVCAR8 have endogenous p53 mutations, whereas OVCAR3 is nonmetastatic in contrast to OVCAR8 that is aggressively metastatic *in vivo.* Human ADSC were purchased from Lonza (Basel, Switzerland) and cultured in MEM-α, nucleosides, GlutaMAX™ medium (MEM) supplemented with FBS, penicillin, and streptomycin. All cells were tested negative for mycoplasma using luciferase reporter assay (Lonza) and authenticated using the short tandem repeat analysis by ATCC (Manassas, VA, USA). CM was prepared when ADSC reached passages 3–6 and 80%–90% confluence. After the medium was changed into fresh complete medium (with or without serum) and culturing for additional 24 h, the medium was collected as ADSC-CM following centrifugation at 300×*g* for 5 min and filtration through 0.22 μm filter and stored at −80°C for the subsequent experiments.

### Characterization of ADSC by Flow Cytometry

ADSC at the third passage were collected by 0.25% trypsin-EDTA (Gibco, Grand Island, NY, USA), washed in PBS and resuspended in PBS at a concentration of 1 × 10^6^ cells/ml. The cells were then blocked with 0.5% bovine serum albumin (BSA) (Sigma-Aldrich, St. Louis, MO, USA) at 4°C for 20 min, followed by additional 30 min incubation at 4°C in the dark with one of the following antibodies: anti-CD44-FITC (1:50), anti-CD73-PE (1:50), anti-CD105-BV421 (1:100), anti-CD31-APC-Cy™7 (1:100), anti-CD45-APC (1:100), and anti-CD90-PerCP-Cy™5.5 (1:50). All antibodies were purchased from BD Biosciences (San Jose, CA, USA). The cells were subsequently washed in 1 ml 0.2% BSA and centrifuged at 500×*g* for 5 min. Antibody binding was detected using a Bio-Rad ZE5 cell analyzer (Bio-Rad, Hercules, CA, USA) and analyzed using the FlowJo^®^ software.

### MTT Assay

OVCAR3 and OVCAR8 cells (2,000 cells/well) were seeded into 96-well plates. After culturing for 24 h, the medium was substituted with fresh complete culture medium (control) or serum containing ADSC-CM. The MTT reagent (10 μl) was added at the end of the indicated time points (1-, 3-, and 5d) and incubated for 4 h, before 100 μl detergent reagent was added. Subsequently, the plates were incubated at room temperature in the dark for 2 h and the absorbance was measured at 570 nm.

### Cell Colony Formation Assay

OVCAR3 and OVCAR8 cells were plated into 6-well plates at the concentration of 400 cells/well and cultured with control medium or serum-containing ADSC-CM for 14 days. The cell colonies were fixed with methanol and stained with crystal violet before being imaged and counted.

### Cell Migration Assay

A modified Transwell™ chamber (BD Falcon, San Jose, CA, USA) with an 8.0-μm pore size was used for the cell migration assay. OVCAR3 and OVCAR8 cells (2 × 10^5^) were suspended in 300 μl of serum-free medium and seeded into the Transwell chamber; control medium or serum-containing ADSC-CM was added into the lower chamber of the 24-well plates. Following 6-h incubation, the cells on the upper side of the chamber membrane were removed with cotton swabs, while the migrated cells on the lower side of the membranes were fixed with methanol and stained with crystal violet. Images were taken at ×10 magnification, and cells in at least three different fields of view were counted.

### Cell Invasion Assay

The 24-well Tumor Invasion System (BD BioSciences, San Jose, CA, USA), which were precoated with Matrigel (BD BioCoat™), were used for cell invasion assay. OVCAR3 or OVCAR8 cells (2 × 10^5^) were seeded in serum-free medium onto upper chamber, and control medium or ADSC-CM were used as a chemoattractant and added into the lower chamber. After incubating for 12 h, the Transwell inserts following fixation with methanol for 20 min were stained with hematoxylin and eosin for 10 min. Pictures were taken at ×10 magnification, and the cells were counted in at least three different fields.

### Measurement of TGF-β in ADSC-CM

The quantitation of TGF-β1 in serum-free ADSC-CM was performed by using Fast Human TGF-β1 ELISA Kit (Tribioscience, Sunnyvale, CA, USA) following the manufacturer’s instructions. ADSC-CM were collected after changing the serum-free medium for 6-, 12-, 24-, and 48 h, respectively.

### Western Blot

OC cells were collected with RIPA buffer (Thermo Scientific, Rockford, IL, USA) containing 1% Halt Proteinase Inhibitor Cocktail (Thermo Scientific). A total of 30 μg protein/lane were loaded onto 10% SDS-PAGE gels and transferred onto PVDF membranes. The blots were blocked with 5% blocking buffer (nonfat milk) at room temperature for 1 h and incubated at 4°C overnight with the primary antibodies against phospho-SMAD2 (p-SMAD2, 1:500 #18338S), SMAD2/3 (1:1,000, #8685S), E-cadherin (1:1,000, #3195S), N-cadherin (1:1,000, #13116S), vimentin (1:1,000, #5741S) (Cell Signaling, Danvers, MA, USA), cytokeratin 7 (1:1,000, #ab181598, Abcam, Cambridge, MA, USA), and GAPDH (1:1,000,#G9545; Sigma-Aldrich, St. Louis, MO, USA). The membranes were washed with PBST and incubated with horseradish peroxidase-conjugated secondary antibodies (1:10,000, goat anti-rabbit, #sc-2004; goat anti-mouse, sc-2005; Santa Cruz, Dallas, TX, USA) at room temperature for 1 h. Protein bands were visualized using chemiluminescence by exposing on X-ray film. The films were scanned and analyzed by Image J.

### SMAD-Dependent Reporter Gene Luciferase Assay

OVCAR3 and OVCAR8 cells were transduced with the lentiviral vector pGF-SMAD2/3/4-mCMV-luciferase-EF1a-puro (System Biosciences, CA) containing SMAD2/3/4 transcriptional response elements (TRE). The medium was changed to control medium (serum-free) or ADSC-CM (serum-free) and incubated for 12 h and treated with 6 ng/ml TGF-β for additional 12 h to activate SMAD2/3/4 pathway. For some groups, the cells were also treated with the TGF-β antagonist at 10 μM SB431542 (Sigma-Aldrich, #S4317) (St. Louis, MO, USA) or vehicle for 24 h. Luciferase activity was measured using Dual-Luciferase Reporter Assay System (Promega, Madison, WI, USA) and normalized by comparing with the control or vehicle-treated group.

### Orthotopic Ovarian Cancer Mouse Model

To track ovarian tumor growth and metastasis *in vivo*, OVCAR 8 cells labeled with luciferase (OVCAR8-Luc2) were used in an orthotopic OC mouse model. 2-month-old immunocompromised NOD.Cg Prkdcscid Il2rgtm1Wjl/SzJ (NSG) female mice (Jackson Laboratory) were intrabursally inoculated with 5 × 10^5^ cells. Then the 20 mice were randomized into four groups 1-week postinoculation and treated with either 1) control medium, 2) serum-free ADSC-CM, 3) SB431542 plus control medium, or 4) SB431542 plus serum-free ADSC-CM *via* peritoneal injection. SB431542 was peritoneally injected with a dose of (10 mg/kg body weight). Mice were treated daily for 5 days a week and for 3 weeks. Tumor growth and metastasis were observed by Xenogen Bioimaging system once a week. All mice were sacrificed at 4 weeks after cell injection. Primary ovarian and metastatic tumors were collected for histology and western blot to determine p-SMAD2 and EMT markers protein levels. All animal experiments were performed in accordance with the protocol approved by the Institutional Animal Care and Use Committee of the University of Tennessee Health Science Center.

### Statistical Analysis

At least three independent experiments in sample triplicate were performed. Data are presented as mean ± SD and Student’s *t*-test was performed using GraphPad Prism 8 (GraphPad, San Diego, CA, USA) to determine significant differences between the treatment and control groups. *p* < 0.05 was considered significant.

## Results

### ADSC Promotes OC Cell Growth, Migration, and Invasion

ADSC were characterized by examining specific marker gene expression using flow cytometry. As shown in **Supplementary Figure S1**, CD44, CD73, CD90, and CD105 are expressed while CD31 and CD45 are absent in ADSC.

**Figure 1:**
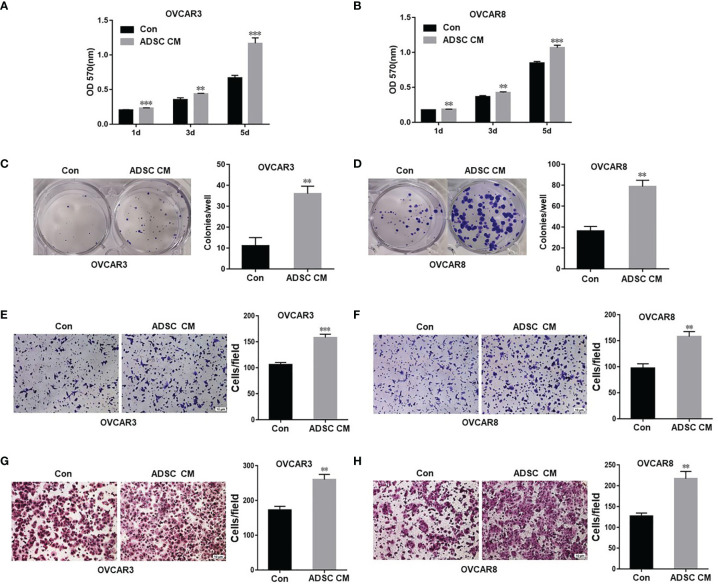
ADSC promote cell growth, migration, and invasion in ovarian cancer cells. **(A, B)** MTT assay was performed to detect the cell proliferation in OVCAR3 **(A)** and OVCAR8 **(B)** cells at different time points, following treatment with ADSC-CM or control medium. **(C, D)** Cell colony formation assay was performed to determine cell survival in OVCAR3 **(C)** and OVCAR8 **(D)** cells treated with ADSC-CM or control medium. **(E, F)** Cell migration in OVCAR3 **(E)** and OVCAR8 **(F)** cells treated with ADSC-CM or control medium performed using Transwell plates. **(G, H)** Cell invasion in OVCAR3 **(G)** and OVCAR8 **(H)** cells treated with ADSC-CM or control medium examined using Matrigel-coated plates. Data represent the mean ± SD of three independent experiments. ^**^ *p* < 0.01, ^***^ *p* < 0.001 compared with the control group.

To assess how ADSC affect OC cell functions, we examined the OC cell proliferation and survival following treatment using ADSC-CM or control medium. ADSC-CM significantly increased OC cell proliferation compared with the control medium at all three time points (1, 3, and 5 days) in both OVCAR3 and OVCAR8 cells (**Figures 1A, B**). ADSC-CM also significantly promote OC cell survival as shown by cell colony formation assay (**Figures 1C, D**).

We also examined cell migration and invasion using the Transwell plates to determine whether ADSC contribute to OC invasiveness and found that ADSC-CM significantly increased cell migration (**Figures 1E, F**) and invasion in both OVCAR3 and OVCAR8 cells (**Figures 1G, H**).

### ADSC Promotes EMT by Activating the TGF-β Signaling Pathway in OC Cells

**Figure 2:**
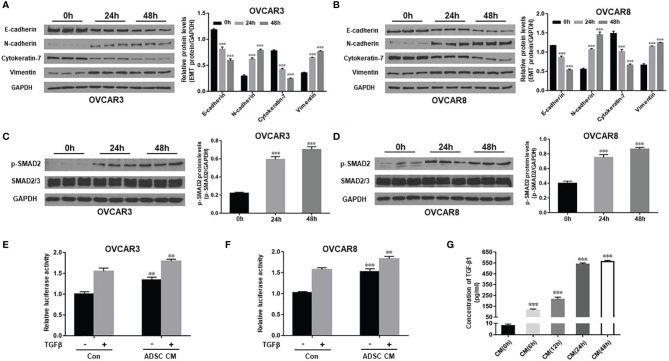
ADSC promotes EMT by activating the TGF-β signaling pathway in OC cells. **(A, B)** Western blot analysis of EMT markers in both OVCAR3 **(A)** and OVCAR8 **(B)** cells following treatment with ADSC-CM for different time durations (0, 24, and 48 h). Right, the quantification analysis of the blots. **(C, D)** Western blot analysis of the p-SMAD2 and SMAD2/3 expression in both OVCAR3 **(C)** and OVCAR8 **(D)** cells following treatment with ADSC-CM for different time durations. Right, the quantification analysis of the blots. **(E, F)** Luciferase activity in OVCAR3 **(E)** and OVCAR8 **(F)** cells following ADSC-CM treatment for 24 h, then 6 ng/ml TGF-β treatment for 12 h. **(G)** ELISA analysis of ADSC-CM collected at different time points to detect the concentrations of TGF-β1. Data represent the mean ± SD of three independent experiments. ^**^ *p* < 0.01, ^***^ *p* < 0.001 compared with the control group.

We have shown previously that TGF-β promotes EMT and contributes to OC tumor cell invasion (32). To understand the mechanisms on how ADSC contribute to OC growth and metastasis, extending our earlier observations, we examined whether ADSC affect EMT phenotypic switch through TGF-β pathway. We determined expression of EMT marker proteins in both OVCAR3 and OVCAR8 following treatment with ADSC-CM for different periods (0, 24, and 48 h). We found that ADSC-CM increased the expression of the mesenchymal markers N-cadherin and vimentin whereas, inhibited the expression of epithelial markers E-cadherin and cytokeratin-7 e in a time-dependent manner in both OC cell lines (**Figures 2A, B**), indicating that ADSC facilitates the EMT of OC cells. Changes in cell morphology are shown in **Supplementary Figure S2**. After treatment with ADSC-CM, some OC cells showed spindle-like shapes.

To explore whether TGF-β signaling pathway is involved in the ADSC-CM-induced EMT, we detected expression of p-SMAD2 and total SMAD2 in OVCAR3 and OVCAR8 cells at different time points after ADSC-CM treatment. We found that ADSC-CM enhanced p-SMAD2 level without changing the total SMAD2 expression (**Figures 2C, D**). We further validated this finding by applying SMAD-dependent reporter gene luciferase assay. ADSC-CM significantly increased SMAD2/3/4 transcriptional activity in both OVCAR3 and OVCAR8 cells (**Figures 2E, F**). Since ADSC-CM activates the TGF-β pathway, we decided to examine whether ADSC indeed secret TGF-β into ADSC-CM using ELISA assay. We found that TGF-β1 in ADSC-CM increased significantly at 6 h, reaching its peak levels between 24 and 48 h (**Figure 2G**). Our data indicate that ADSC promotes EMT phenotypic switch by activating the TGF-β pathway in OC cells.

### Inhibition of TGF-β Pathway Blocks ADSC-Induced OC Cell Survival, Migration, and Invasion

**Figure 3:**
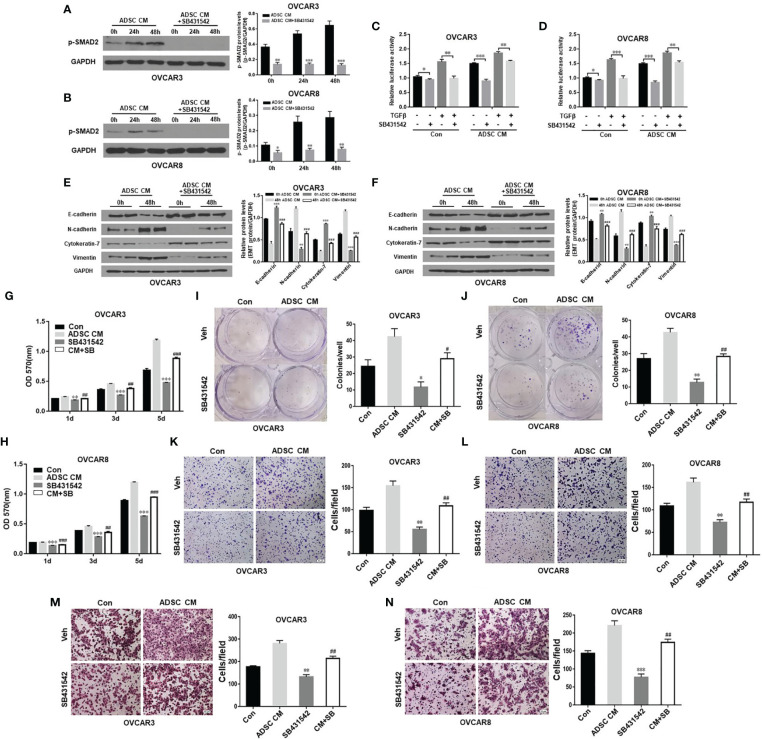
Inhibition of TGF-β pathway antagonizes ADSC-induced OC growth, migration, and invasion. **(A, B)** Western blot analysis of the p-SMAD2 expression in both OVCAR3 **(A)** and OVCAR8 **(B)** cells treated with 10 μM SB431542 for 24 h and then ADSC-CM for different time durations. Right, the quantification analysis of the blots. **(C, D)** Luciferase activity in OVCAR3 **(C)** and OVCAR8 **(D)** cells following ADSC-CM treatment for 24 h, SB431542 treatment for 24 h, then 6 ng/ml TGF-β treatment for 12 h. **(E, F)** Western blot analysis of EMT markers in both OVCAR3 **(E)** and OVCAR8 **(F)** following treatment with ADSC-CM and SB431542 (10 μM) for 48 h. Right, the quantification analysis of the blots. **(G, H)** MTT assay was performed to detect the cell proliferation ability of OVCAR3 **(G)** and OVCAR8 **(H)** cells at different time points following ADSC-CM and SB431542 (10 μM) treatment. **(I, J)** Cell colony formation assay determined cell survival in OVCAR3 **(I)** and OVCAR8 **(J)** cells following ADSC-CM and SB431542 (5 μM) treatment. **(K**, **L)** Cell migration in OVCAR3 **(K)** and OVCAR8 **(L)** cells treated with ADSC-CM or control medium following SB431542 (10 μM) treatment 24 h performed using Transwell plates. **(M, N)** Cell invasion in OVCAR3 **(M)** and OVCAR8 **(N)** cells treated with ADSC-CM or control medium following SB431542 (10 μM) treatment for 24 h using Matrigel-coated plates. Data represent the mean ± SD of three independent experiments. ^*^ *p* < 0.05, ^**^ *p* < 0.01, ^***^ *p* < 0.001 compared with the corresponding control group; ^#^ *p* < 0.05, ^##^ *p* < 0.01, ^###^ *p* < 0.001 compared with the corresponding ADSC-CM group.

Adipocytes in omentum contribute to OC metastasis. ADSC can differentiate into multiple cancer-associated cell types including adipocytes, fibroblasts, and endothelial cells. Targeting ADSC in omentum as major cell type in OC TME has great therapeutic potentials. Therefore, we tested whether inhibition of TGF-β pathway activated by ADSC affects OC function. We treated OVCAR3 and OVCAR8 cells with 10 μM TGF-β receptor inhibitor SB431542 for 24 h and then added ADSC-CM into culture for different time points (0, 24, and 48 h). ADSC activation of SMAD2 phosphorylation in OC cells was significantly inhibited by SB431542 (**Figures 3A, B**). We also validated this finding using SMAD-dependent reporter gene luciferase assay. Similarly, ADSC-induced SMAD2/3/4 transcriptional activity was significantly inhibited by SB431542 treatment (**Figures 3C, D**). Next, we tested whether SB431542 treatment of the OC cells affected the ADSC-CM-induced EMT phenotypic switch. We found that SB431542 significantly reduced EMT in OC cells induced by ADSC as shown by the downregulation of mesenchymal markers and upregulation of epithelial markers compared with vehicle-treated cells (**Figures 3E, F**). In addition, ADSC-induced OC cell proliferation, cell colony formation, migration, and invasion were also significantly inhibited by SB431542 (**Figures 3G** **–N**). Our results indicate that targeting the TGF-β pathway can effectively inhibit ADSC-induced OC cell growth, migration, and invasion.

### ADSC Promotes Primary Tumor Growth and Metastasis in an Orthotopic OC Mouse Model

**Figure 4:**
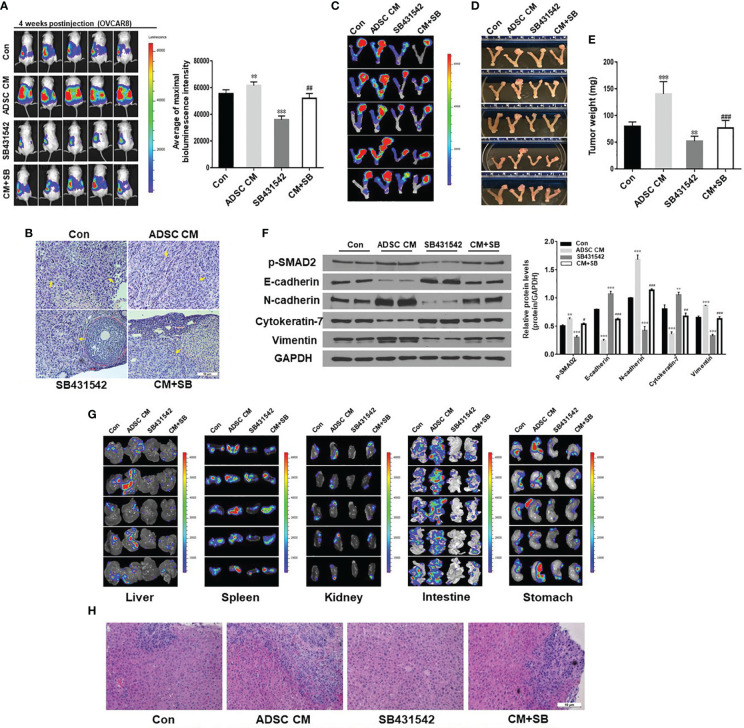
ADSC enhance primary ovarian tumor growth and metastasis in an orthotopic ovarian cancer mouse model. **(A)** Live animal imaging of primary tumors in ovaries of mice at 4** **weeks following intrabursal injection of OVCAR8 cells. Right, the quantitative bioluminescence intensities. **(B)** H&E staining of primary tumor in ovaries. There are high-grade serous carcinomas involving the ovarian stroma. No normal ovary structure could be seen in the control and ADSC-CM groups. Tumors are indicated by yellow arrows. **(C)** Primary tumors in ovaries of mice were identified by live animal imaging. **(D, E)** Tumors in ovaries of mice were dissected, imaged **(D)**, and weighed **(E)**. **(F)** Western blot analysis of p-SMAD2 and EMT markers in primary ovarian tumors. Right, the quantification analysis of the blots. **(G)** Metastatic tumors in liver, spleen, kidney, intestine, and stomach of xenografted mice were identified by live animal imaging. **(H)** H&E staining of metastatic tumors in livers of xenografted mice. Data represent the mean ± SD of three independent experiments. ^**^ *p* < 0.01, ^***^ *p* < 0.001 compared with the control group. ^#^ *p* < 0.05, ^##^ *p* <** **0.01, ^###^ *p* < 0.001 compared with the corresponding ADSC-CM group.

To test whether ADSC promote ovarian tumor progression and metastasis *in vivo* using the orthotopic OC mouse model we described before (33), we intrabursally injected OVCAR8 cells labeled with luciferase into immunocompromised NSG female mice, and then treated mice with ADSC-CM and SB431542. OC primary tumors and metastases were significantly higher in mice treated with ADSC-CM compared with control medium. Moreover, progression of the primary tumors was significantly lower in mice treated with SB43152 compared with vehicle as shown by bioluminescent intensity (**Figure 4A**). Histologic examination confirmed OC growth in the ovaries of the mice (**Figure 4B**). All 4 groups showed malignant epithelial tumor with serous differentiation, nest, or solid/glandular growth and moderate to severe nuclear atypia. Cytologically, tumor cells showed prominent nuclear atypia, characterized by highly pleomorphic, irregular nuclear contours, and variably conspicuous nucleoli.

Consistent with bioluminescence results, a significant difference in tumor weight among four groups was observed (**Figures 4C** **–E**). ADSC-CM significantly stimulated tumor growth in ovaries, and ADSC-CM-induced tumor growth was significantly suppressed by SB431542. We also examined p-SMAD2 and EMT markers in primary ovarian tumors using Western blot. ADSC-CM treatment increased expression of p-SMAD2, N-cadherin, and vimentin and reduced the expression of E-cadherin and cytokeratin-7 (**Figure 4F**). ADSC-induced EMT marker expression was significantly inhibited by SB431542 treatment (**Figure 4F**). We then examined tumor metastasis in multiple peritoneal organs and tumors were found in liver, spleen, kidney, intestine, and stomach (**Figures 4G, H**). ADSC-CM treatment significantly promoted tumor dissemination in these organs, which was significantly reduced by SB431542 treatment. Taken together, our results show that ADSC-CM significantly enhances primary ovarian tumor growth and metastasis by promoting EMT and activating the TGF-β pathway in an orthotopic OC mouse model.

## Discussion

In the present study, we investigated whether ADSC contributes to OC growth and metastasis by using the transfer of ADSC-CM. Our data indicate that ADSC-CM promotes OC growth and metastasis at least partially *via* promoting EMT and activating the TGF-β pathway. ADSC-CM enhances OC cell proliferation, migration, and invasion. Consistent with the role of TGF-β, pharmacological inhibition of TGF-β signaling pathway antagonizes the effects of ADSC-CM on OC function and tumor metastasis. These findings indicate that ADSC-derived TGF-β is a major regulator of OC TME, which contributes to OC tumor progression and metastasis. These findings strongly suggest that TGF-β signaling can be targeted to improve the therapeutic outcomes in OC patients.

Previous studies showed that ADSC play important roles in the interaction between TME and tumor progression by secreting soluble factors and exosomes, including breast cancer, head and neck cancer, and osteosarcoma (34–38). Coculture with ADSC or treatment with ADSC-CM increased metalloproteinase (MMP) 2/9 expression and activated STAT3 transcription factor in osteosarcoma cells, therefore promoted osteosarcoma cell proliferation and invasion (35). Human ADSC secreted CSCL1/8 in contacting with breast cancer cells and then promoted angiogenesis and tumor growth of breast cancer (36). Coculture of human ADSC and head/neck cancer cells showed elevated MMP 2/9 expression and stimulated migration of cancer cells but had no effect on cancer cell growth (38). ADSC from omentum of OC cancer patients induced OC cell growth, migration, and chemoresistance (17). Another study reported that coculture of omentum ADSC and OC cells promoted OC cell proliferation and invasion *via* increased MMP secretion of cancer cells (18). Proteomic analysis of OC cells after treatment with ADSC-CM identified TMSB4X as a factor that mediates protumor effect of ADSC (20). Our findings are consistent with the previous reports that ADSC-CM enhances growth and metastasis of OC both *in vitro* and *in vivo* (18, 20). However, here we show an additional and different molecular mechanism by demonstrating that ADSC-derived TGF-β activates EMT, which in turn contributes to OC progression and metastasis.

We show here that ADSC promotes EMT in OC cells. Consistent with this study, ADSC-CM has also been shown to stimulate EMT in glioma cells (29), lung cancer cells (30), and breast cancer cells (31), suggesting that the ADSC may play a pivotal and general role in tumor metastasis by promoting EMT. EMT is well-known to be associated with tumor metastasis and chemoresistance (39, 40). Our findings indicate that ADSC can also promote OC growth and metastasis through stimulation of EMT. We previously showed that TGF-β promotes EMT in OC (32). Here, we detected TGF-β secreted from cultured ADSC by ELISA assay, which can activate TGF-β signaling in the OC cells. Using a TGF-βR½ inhibitor, SB431542, we show for the first time that blocking of TGF-β pathway attenuates the promotion effects of ADSC on the OC growth and metastasis *in vitro* and *in vivo*. Thus, our findings demonstrate that ADSC can promote OC progression through promoting EMT *via* activation of TGF-β signaling.

Although we demonstrated that ADSC-CM promote OC progression and metastasis by activating the TGF-β pathway, there are multiple growth factors secreted from ADSC and multiple signaling pathways might be involved in the interaction between ADSC and OC. It is essential to identify those growth factors or adipokines secreted from ADSC through the proteomic approach to further understand the importance of ADSC in OC metastasis. In addition, we tested the therapeutic effects of TGF-β inhibitor in OC mouse models; however, it is important to further test whether TGF-β inhibitor has synergistic effects in enhancing the chemotherapy drugs in OC mouse models.

In conclusion, ADSC residing in the TME contributes to OC growth and metastasis, at least in part through promoting EMT and activating the TGF-β signaling. These findings not only help us understand the importance of ADSC in the TME of OC but also provide experimental evidence to target ADSC in TME for the treatment of OC and potentially of several other aggressive cancers.

## Data Availability Statement

The original contributions presented in the study are included in the article/**Supplementary Material**. Further inquiries can be directed to the corresponding authors.

## Ethics Statement

The animal study was reviewed and approved by the Institutional Animal Care and Use Committee (IACUC) at the University of Tennessee Health Science Center.

## Author Contributions

XL, GZ, and XH performed experiments. WZ and JY designed experiments and wrote the manuscript. WZ, JY, GT, YW, and B-MZ edited the manuscript. All authors read and approved the final version.

## Funding

This study was supported by a grant 1R21CA216585-01A1 from NCI and a CORNET Award of UTHSC to YJ and a grant CA-092160-21 from NCI to TG.

## Conflict of Interest

The authors declare that the research was conducted in the absence of any commercial or financial relationships that could be construed as a potential conflict of interest.

## Publisher’s Note

All claims expressed in this article are solely those of the authors and do not necessarily represent those of their affiliated organizations, or those of the publisher, the editors and the reviewers. Any product that may be evaluated in this article, or claim that may be made by its manufacturer, is not guaranteed or endorsed by the publisher.
