# Supplementary material for: Adipose-Derived Stem Cells Facilitate Ovarian Tumor Growth and Metastasis by Promoting Epithelial to Mesenchymal Transition Through Activating the TGF-β Pathway
Source: Front Oncol. 2021 Dec 22;11:756011. doi: 10.3389/fonc.2021.756011 (PMC8727693; doi:10.3389/fonc.2021.756011)
Supplement: Supplementary file 1 [file DataSheet_1.pdf]

Supplementary Material

Supplementary Figure

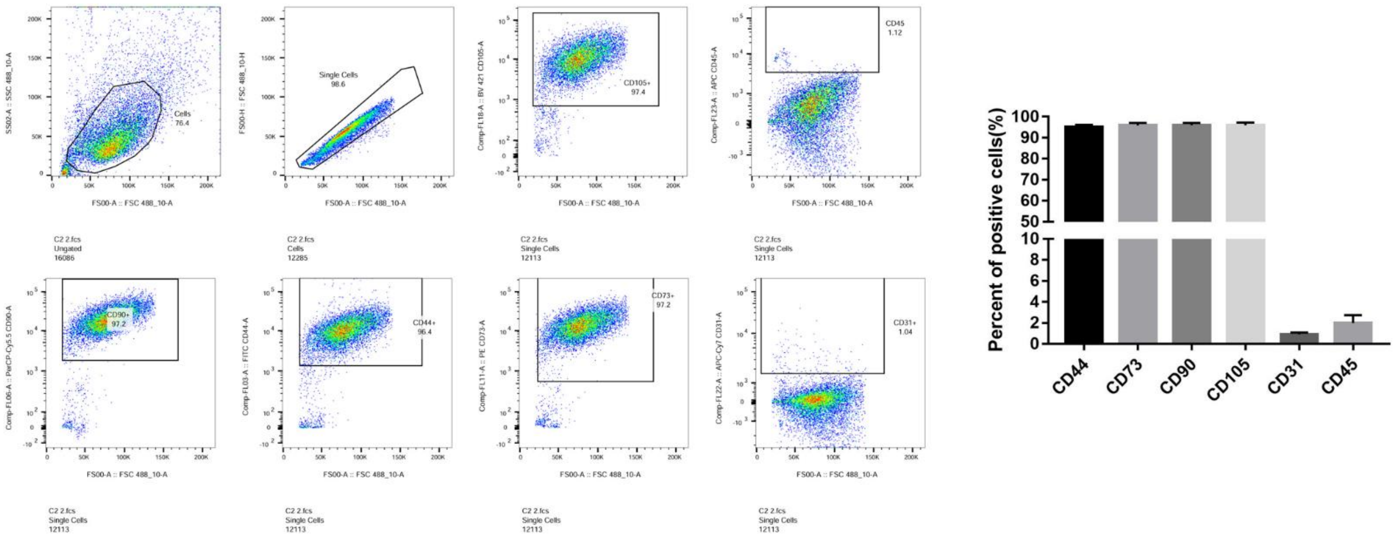

Supplementary Figure 1. The surface markers of ADSC were identified by flow cytometry. ADSC were positive for CD44, CD73, CD90, and CD105 and negative for CD31 and CD45.

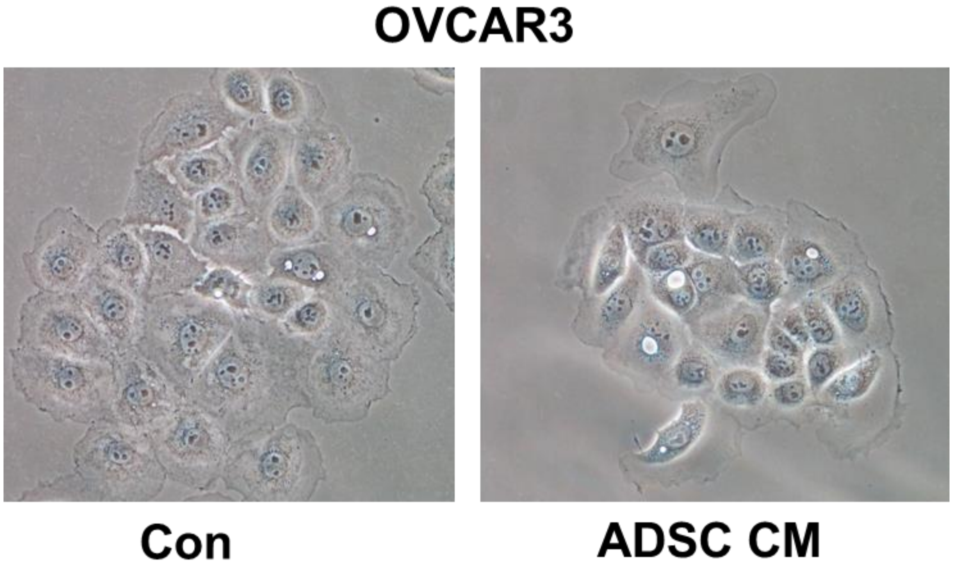

Supplementary Figure 2. Morphology of OVCAR3 before and after treatment with ADSC CM.
