# Supplementary material for: Adipose-Derived Stem Cells Facilitate Ovarian Tumor Growth and Metastasis by Promoting Epithelial to Mesenchymal Transition Through Activating the TGF-β Pathway
Source: Front Oncol. 2021 Dec 22;11:756011. doi: 10.3389/fonc.2021.756011 (PMC8727693; doi:10.3389/fonc.2021.756011)

Figure 1

C Colone formation (OV3)

control(MEM serum)

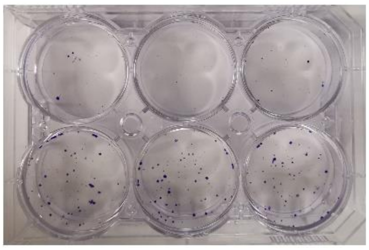

ADSC CM (serum)

D Colone formation (OV8)

control (MEM serum)

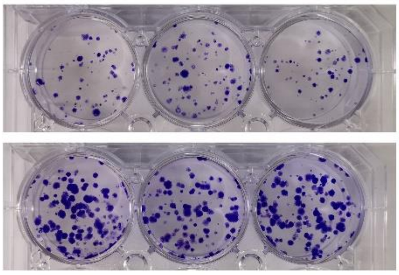

ADSC CM (serum)

E Migration (OV3)

CON

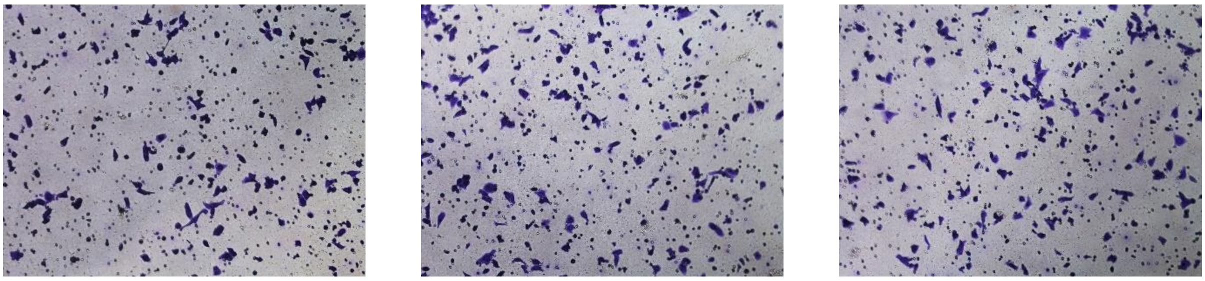

ADSC CM

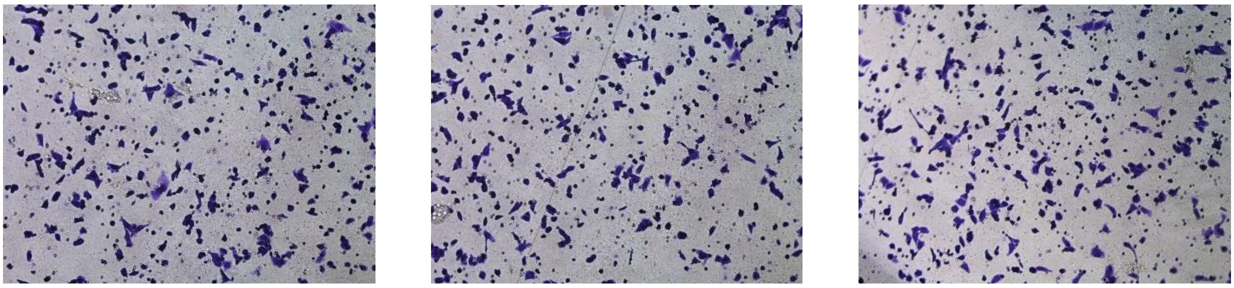

F Migration (OV8)

CON

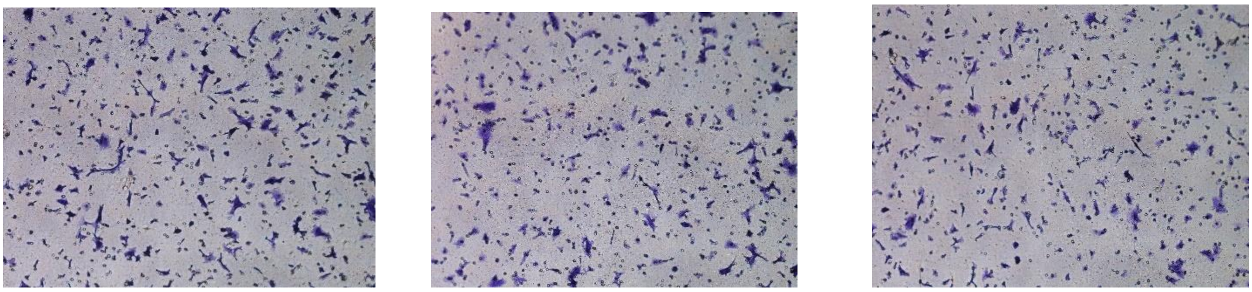

ADSC CM

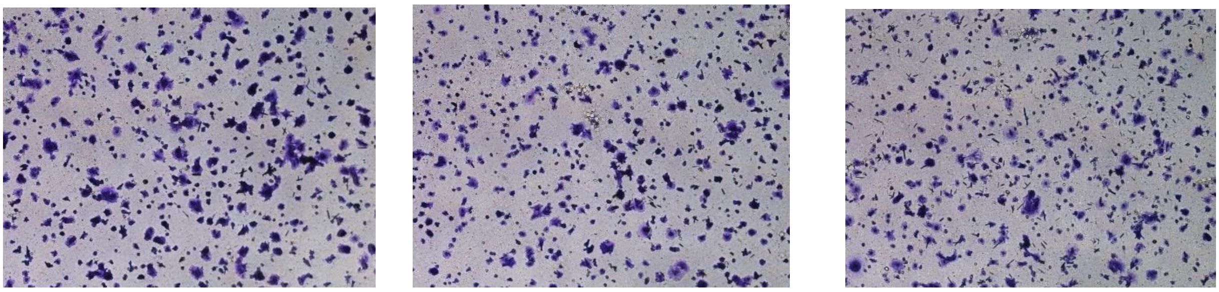

G Invation (OV3)

CON

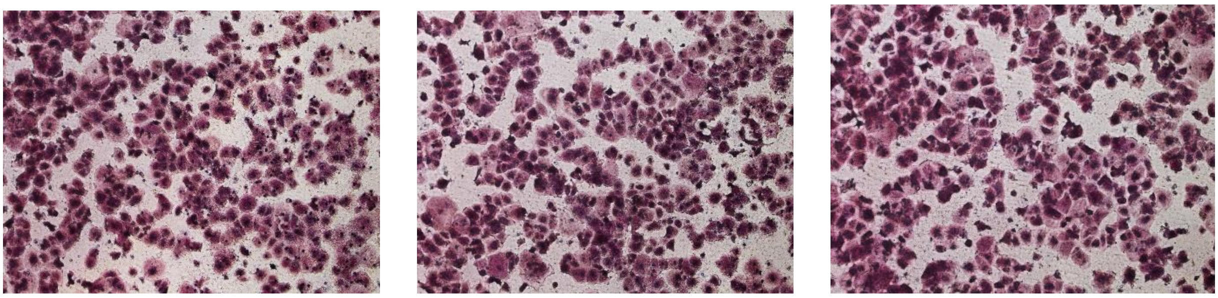

ADSC CM

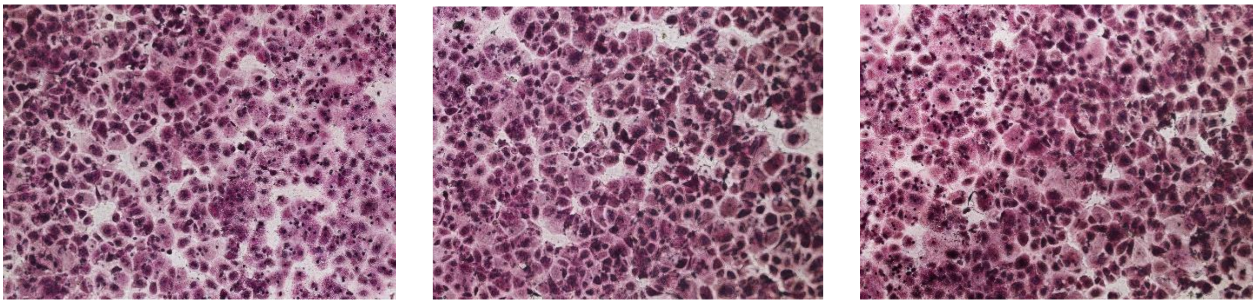

H Invation (OV8)

CON

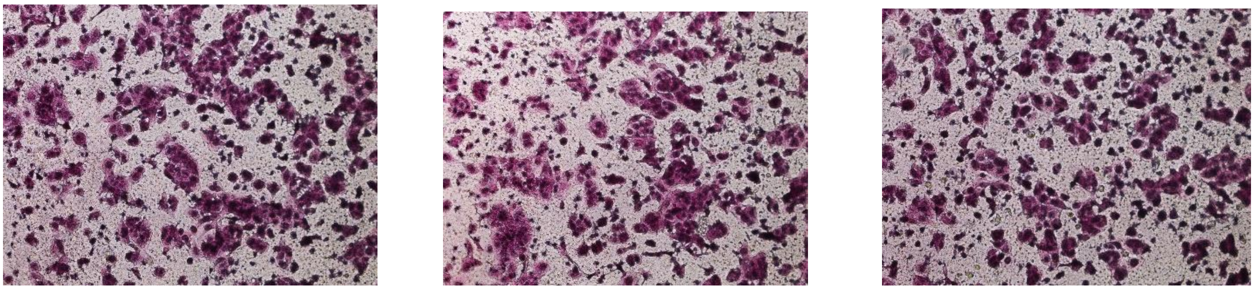

ADSC CM

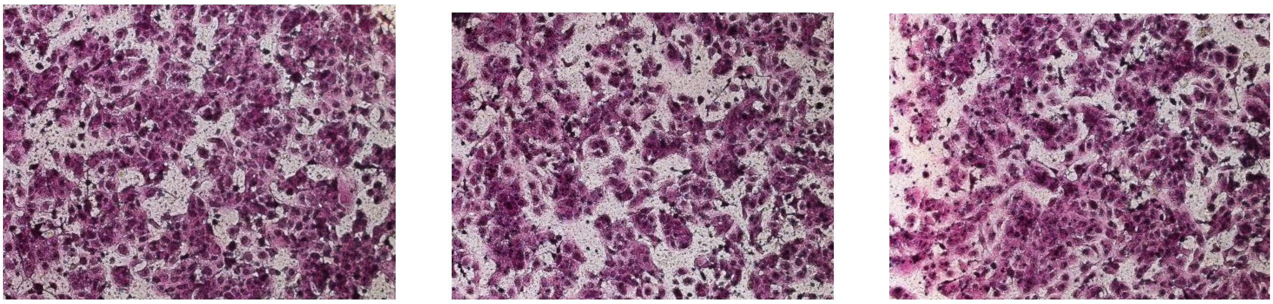

Figure 2

A EMT (OV3)

E-cad

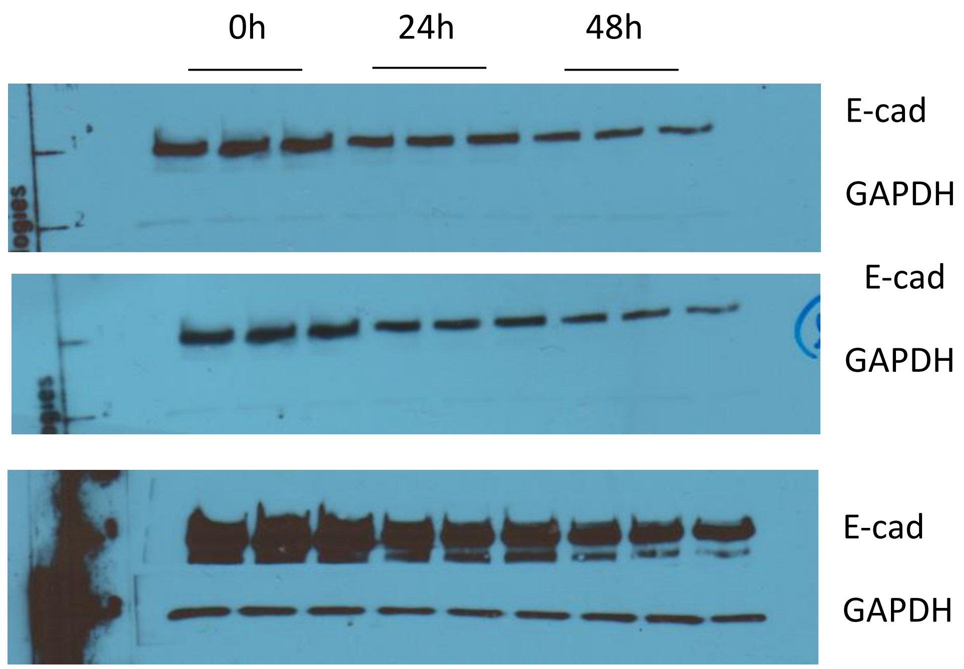

N-cad

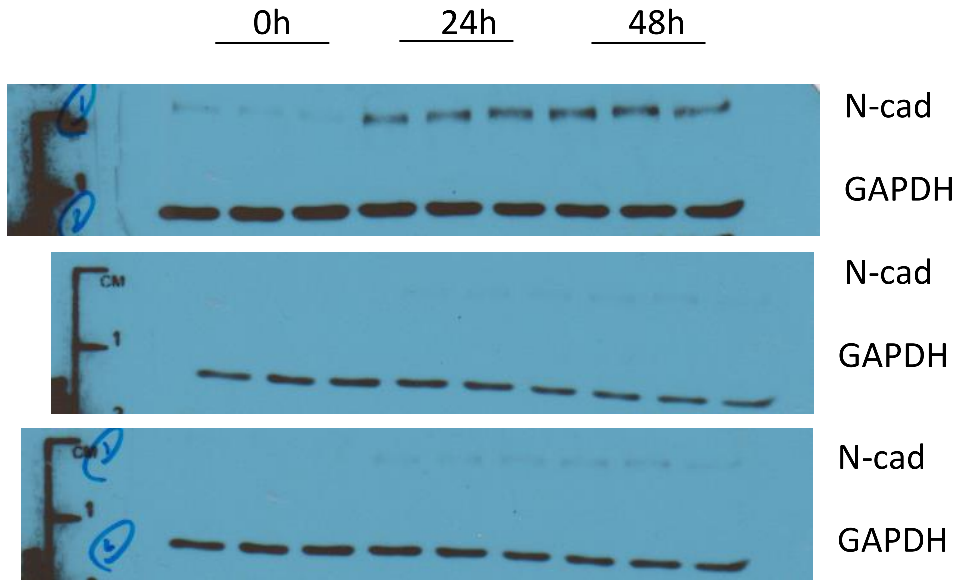

Cyt7

0h 24h 48h

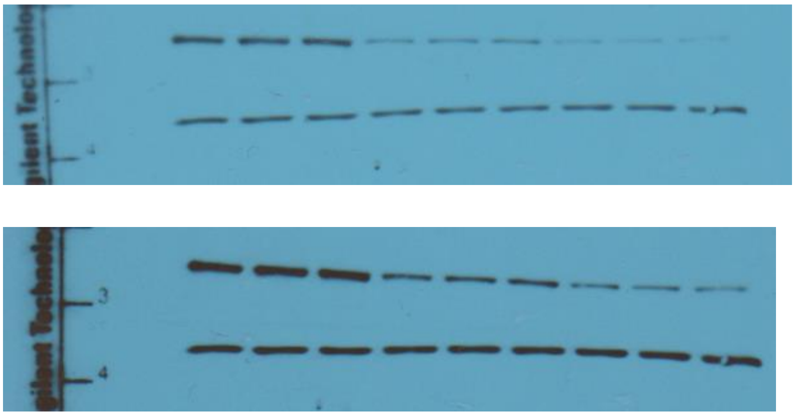

Cyt-7
GAPDH

Cyt-7
GAPDH

Vimentin

0h 24h 48h

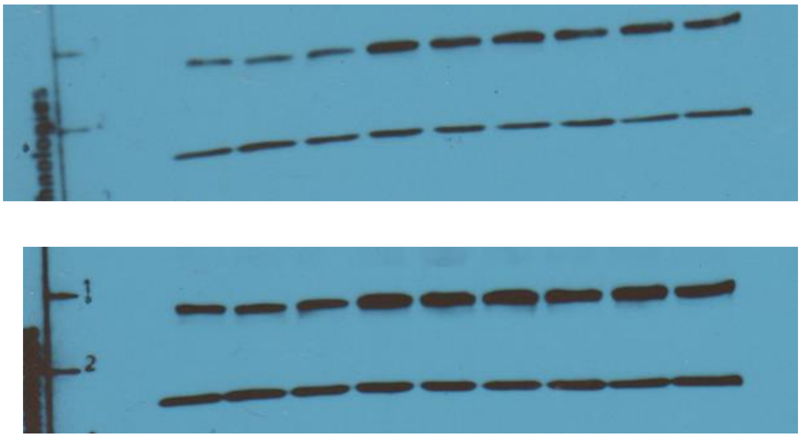

Vimentin
GAPDH

Vimentin
GAPDH

B EMT (OV8)

E-cad

0h 24h 48h

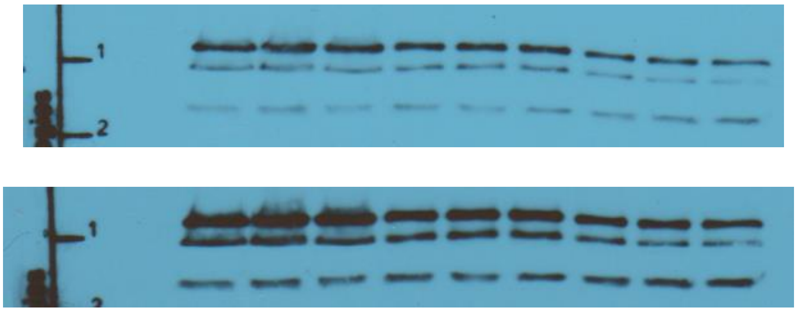

E-cad
GAPDH

E-cad
GAPDH

N-cad

0h 24h 48h

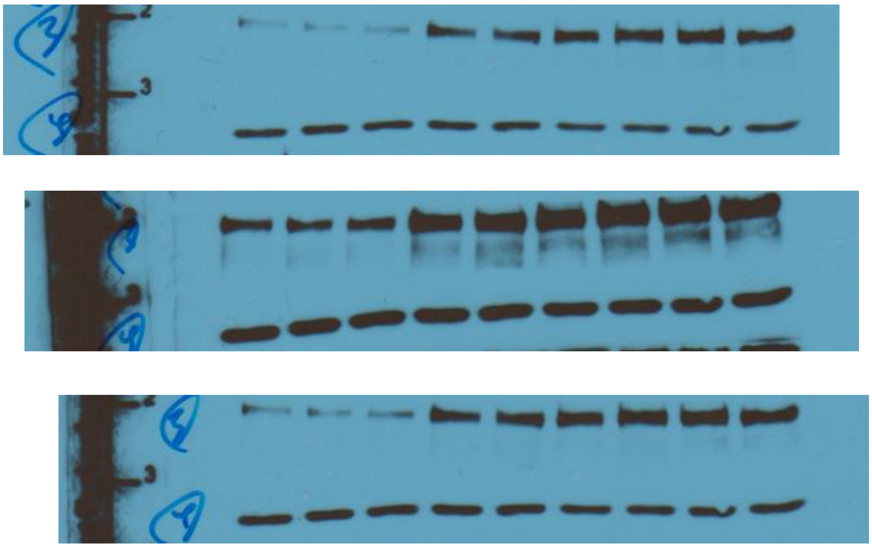

N-cad

GAPDH

N-cad

GAPDH

N-cad

GAPDH

Cyt7

0h 24h 48h

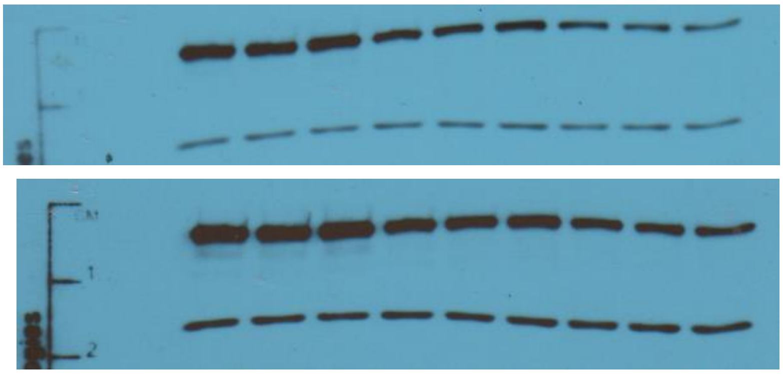

Cyt-7

GAPDH

Cyt-7

GAPDH

Vimentin

0h 24h 48h

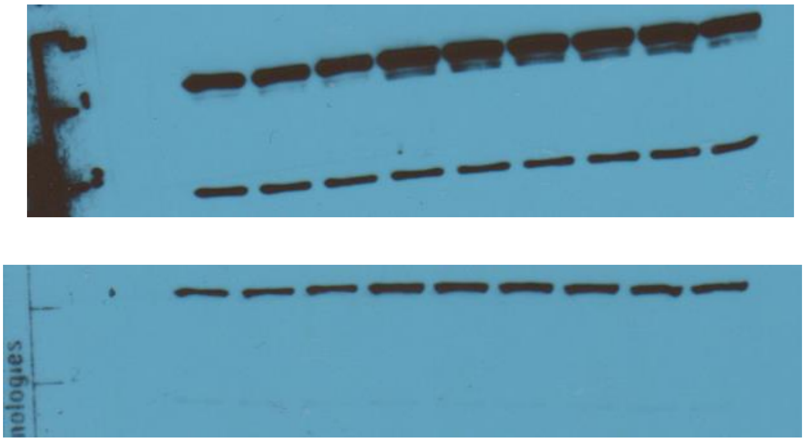

Vimentin

GAPDH

Vimentin

GAPDH

C p-SMAD2 (OV3)

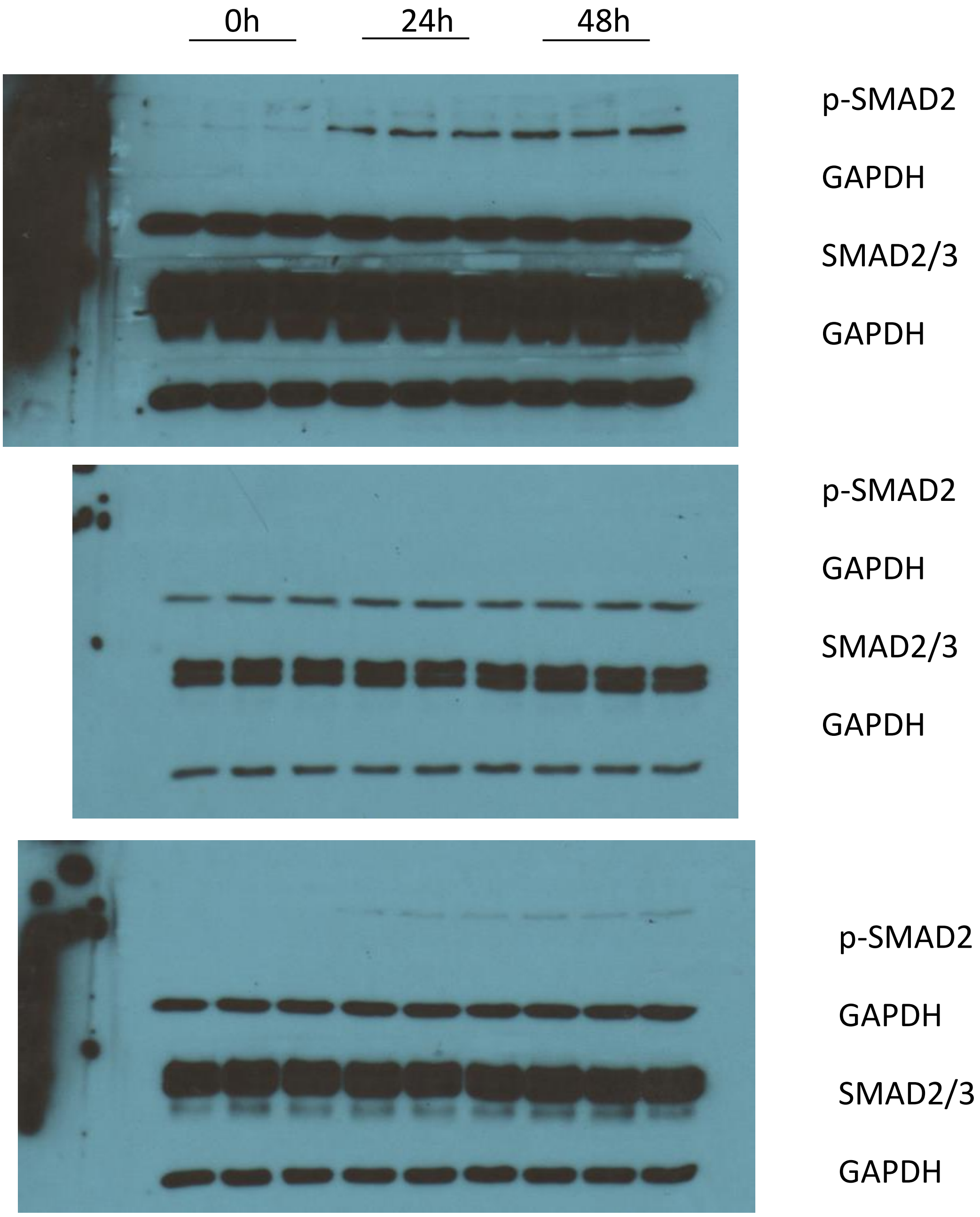
0h
24h
48h
p-SMAD2
GAPDH
SMAD2/3
GAPDH
p-SMAD2
GAPDH
SMAD2/3
GAPDH
p-SMAD2
GAPDH
SMAD2/3
GAPDH

D p-SMAD2 (OV8)

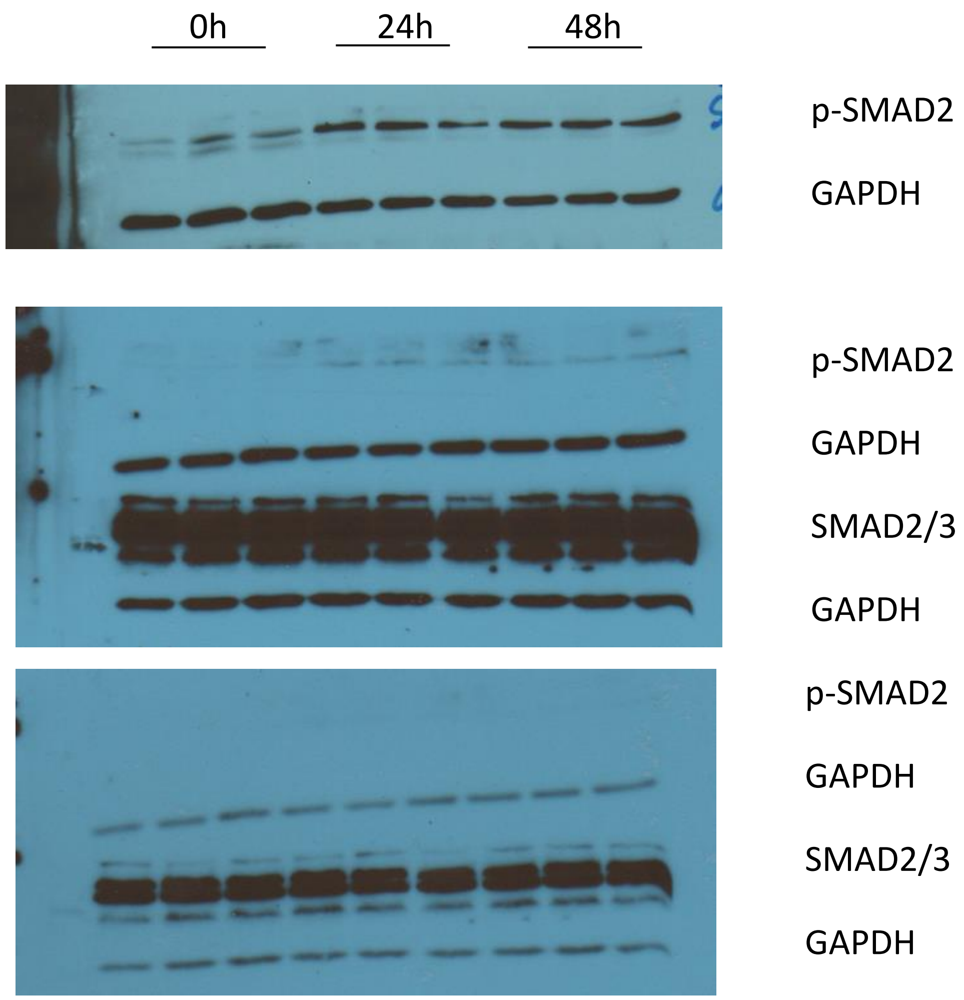
0h
24h
48h
p-SMAD2
GAPDH
p-SMAD2
GAPDH
SMAD2/3
GAPDH
p-SMAD2
GAPDH
SMAD2/3
GAPDH

Figure 3

A OV3 p-SMAD2(CM+SB)

ADSC CM ADSC CM+SB

0h 24h 48h 0h 24h 48h

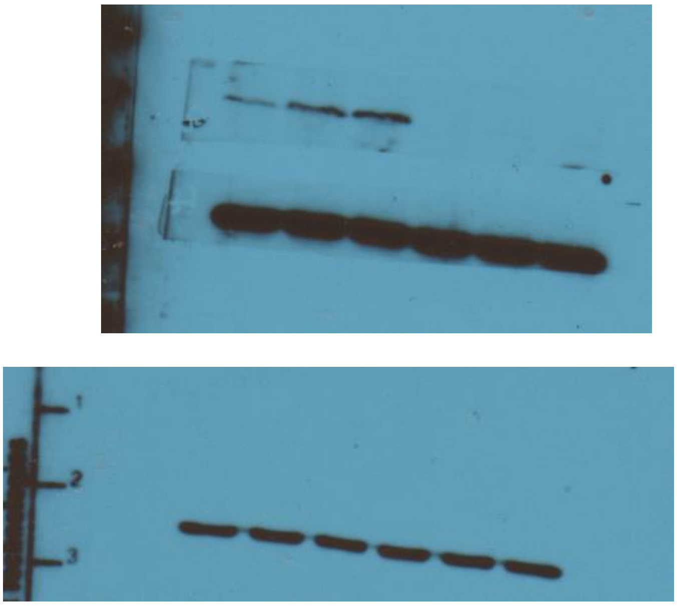

p-SMAD2

GAPDH

p-SMAD2

GAPDH

B OV8 p-SMAD2(CM+SB)

ADSC CM ADSC CM+SB

0h 24h 48h 0h 24h 48h

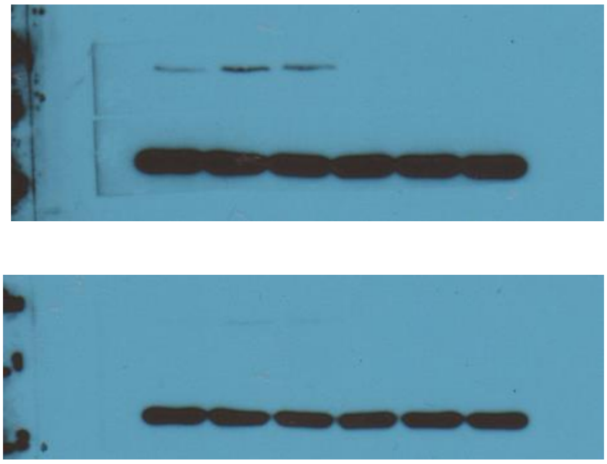

p-SMAD2

GAPDH

p-SMAD2

GAPDH

E OV3 EMT (CM+SB)

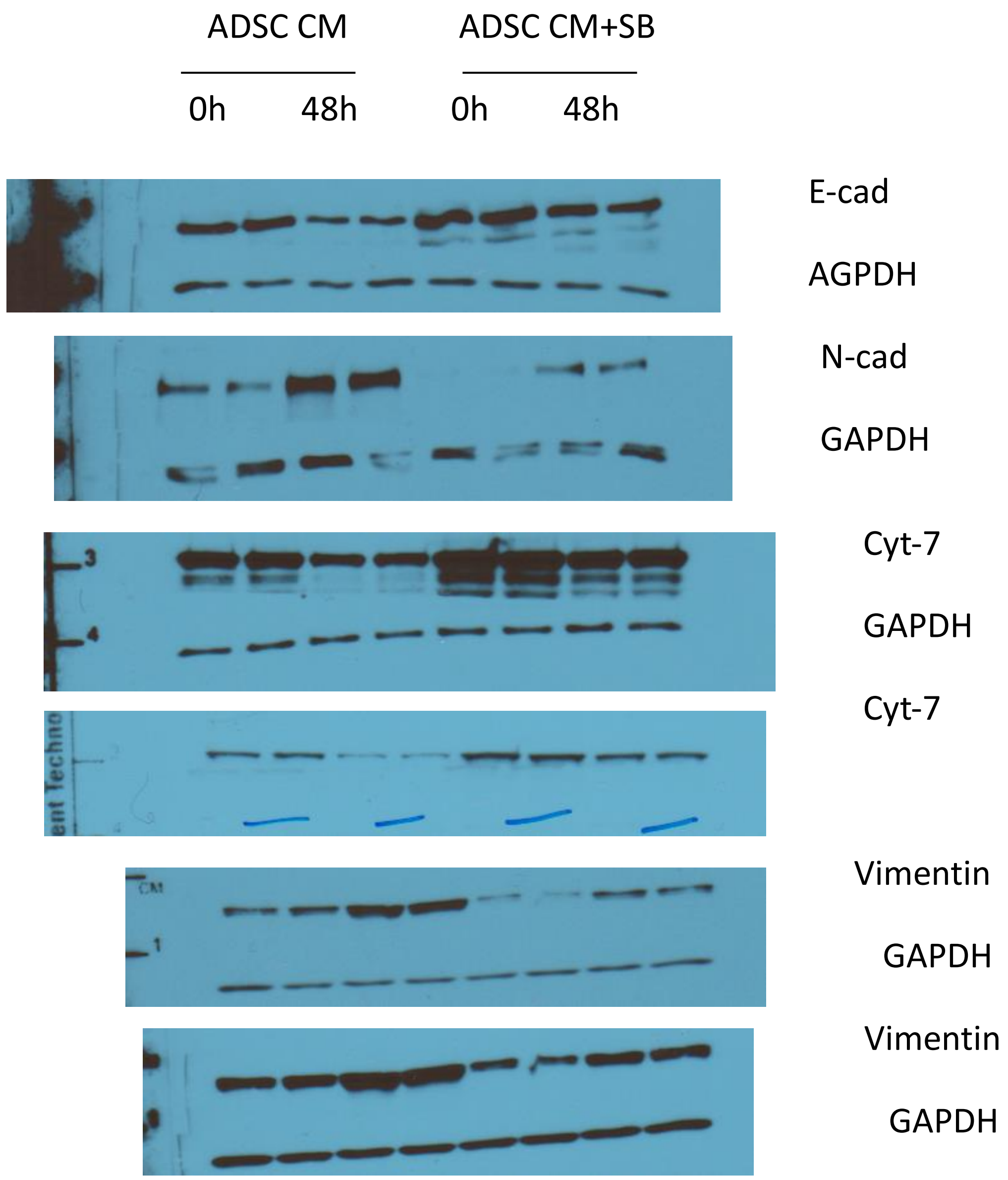

F OV3 EMT (CM+SB)

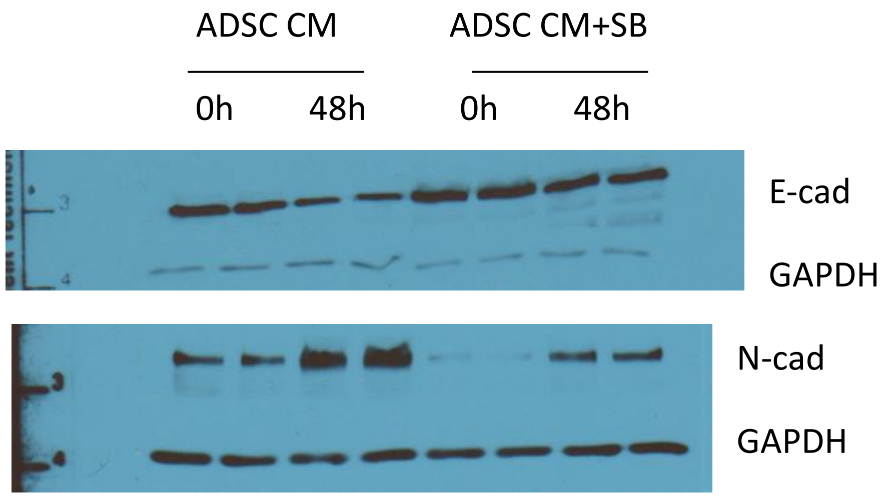

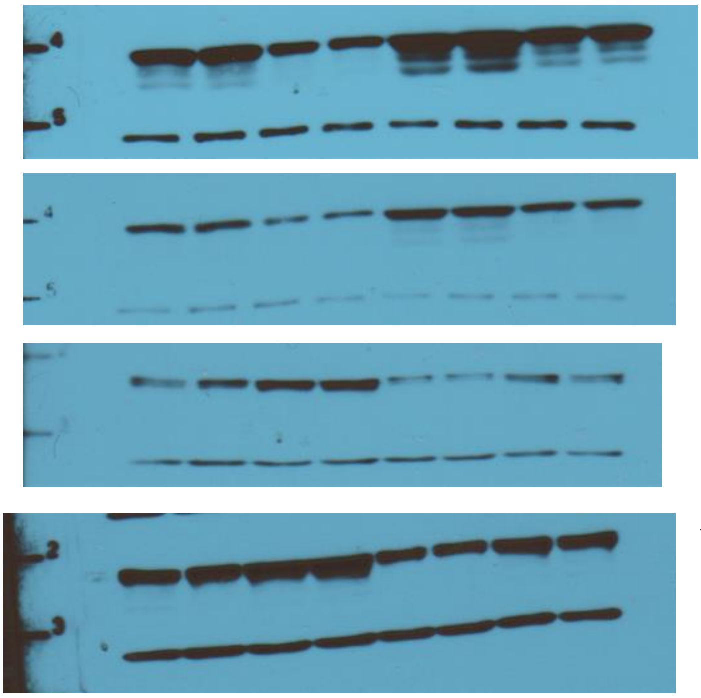

Cyt-7

GAPDH

Cyt-7

GAPDH

Vimentin

GAPDH

Vimentin

GAPDH

I Colone formation (OV3)   (CM+SB)

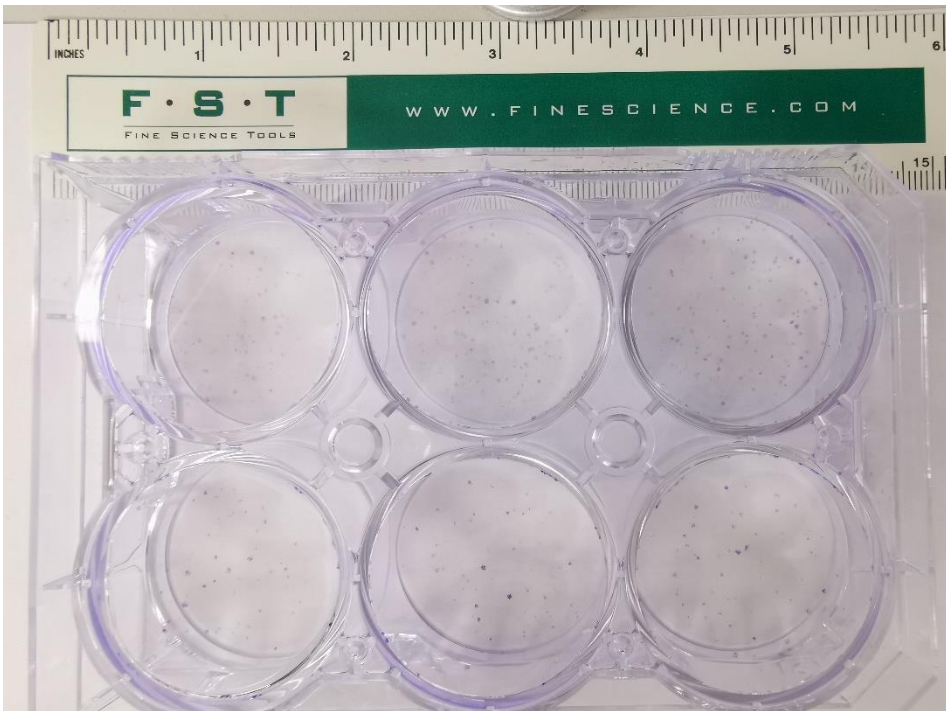

ADSC CM

Con

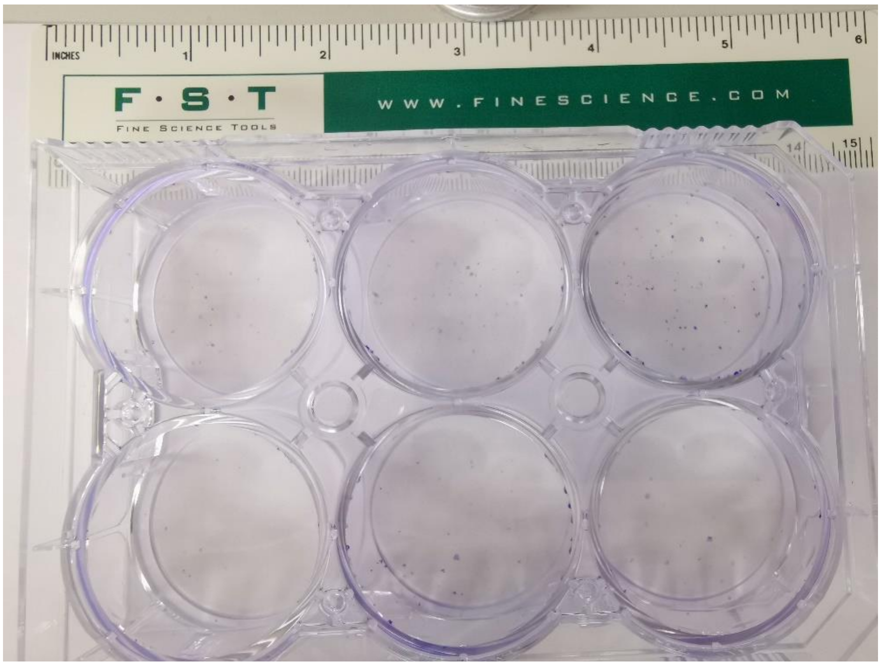

ADSC CM+SB

Con+SB

J Colone formation (OV8) (CM+SB)

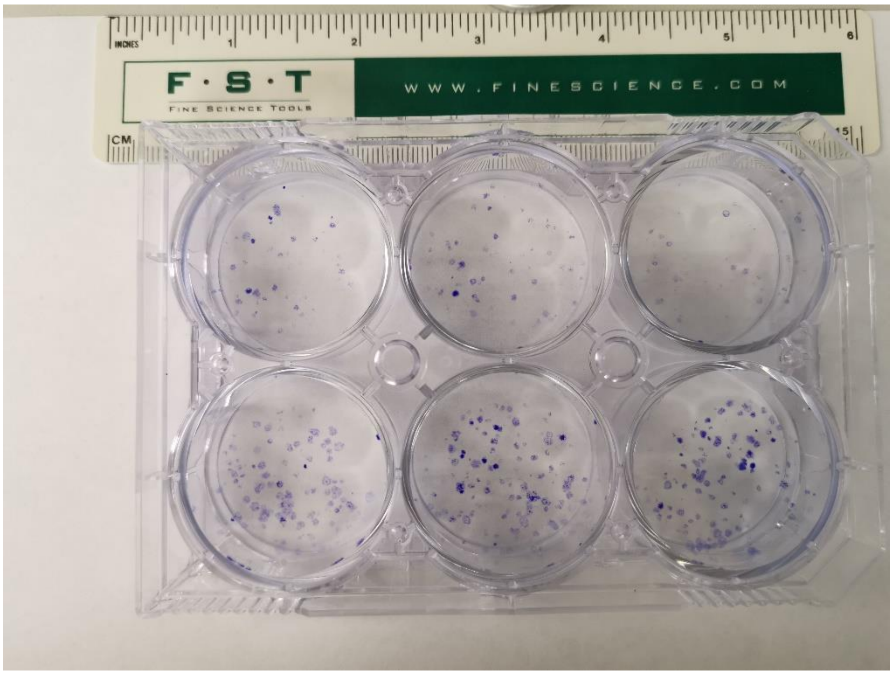

Con+SB

Con

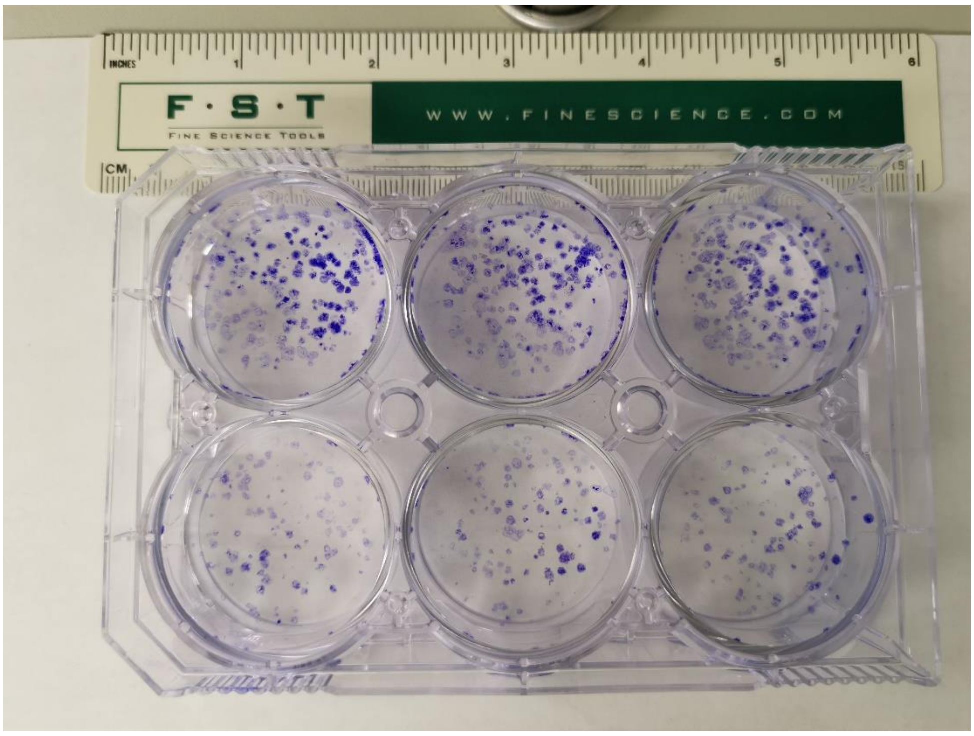

ADSC CM

CM+SB

K Migration (OV3)(CM+SB)

Con

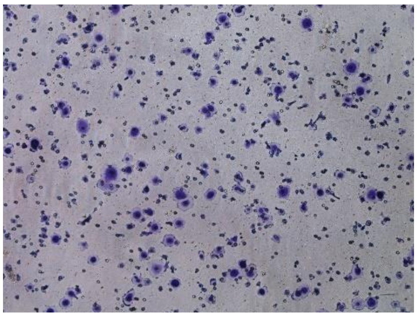
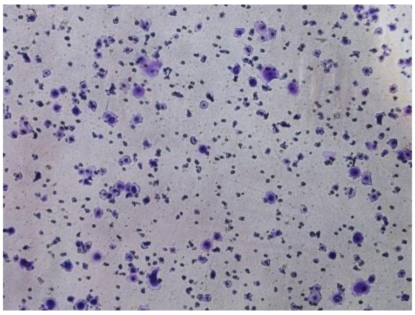
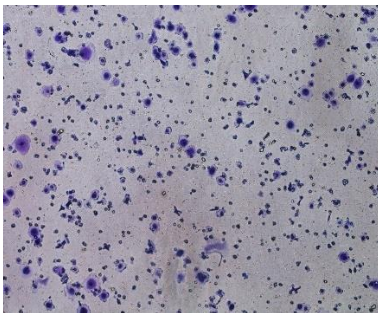

ADSC CM

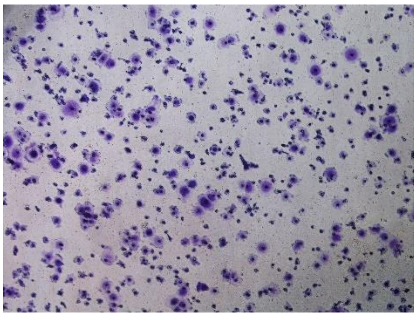
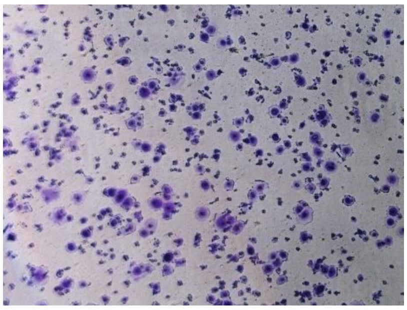
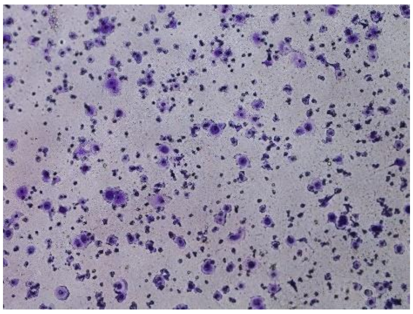

Con+SB

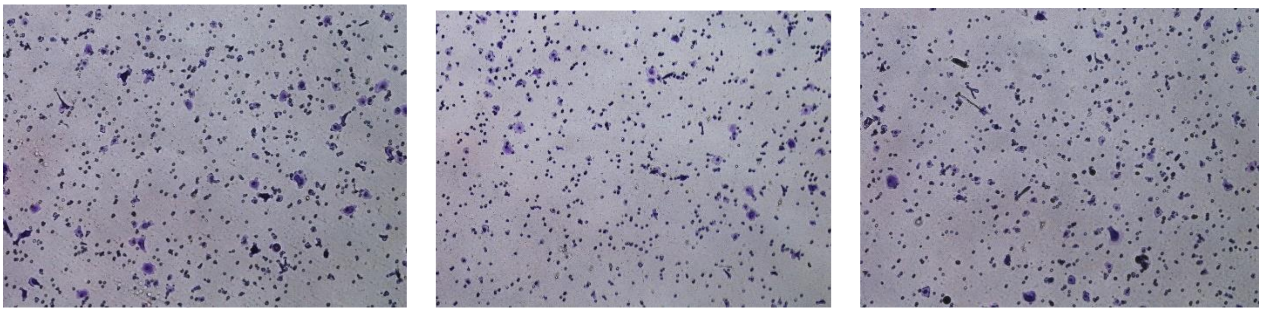

ADSC +CM

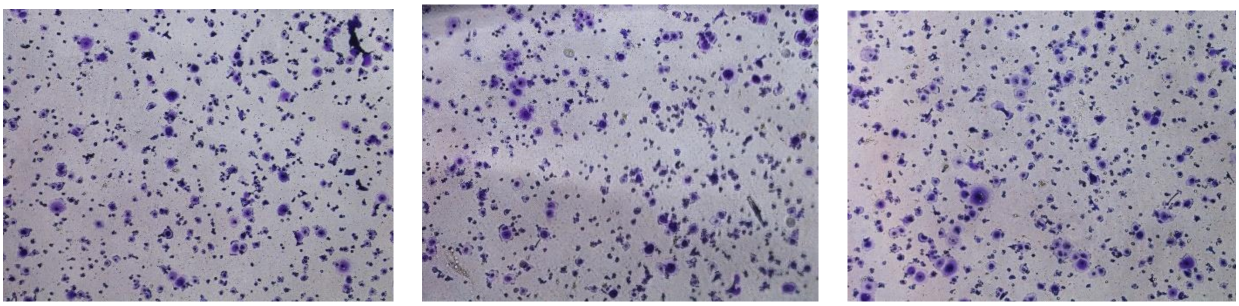

L Migration (OV8)(CM+SB)

Con

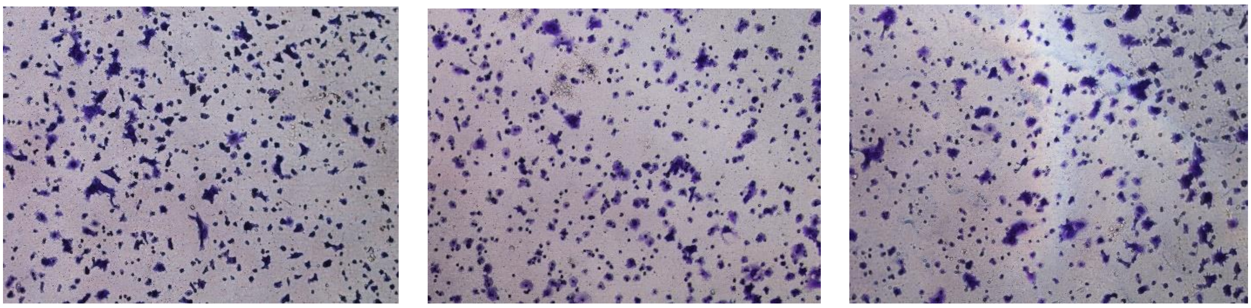

ADSC CM

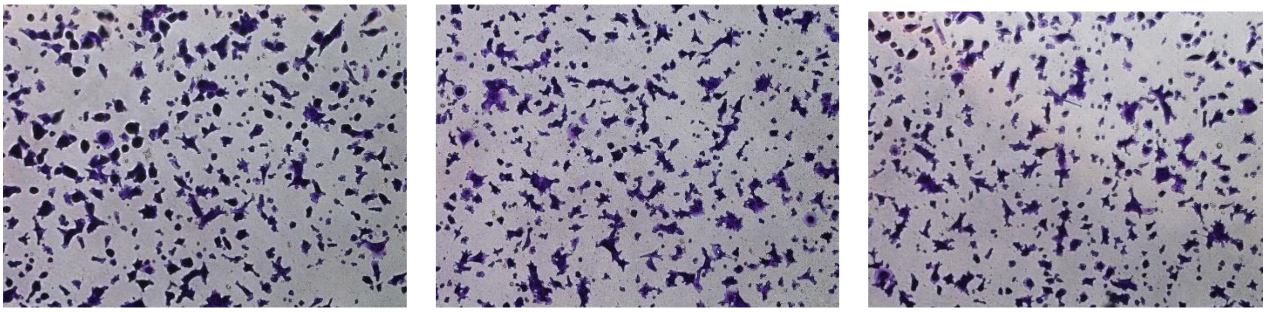

Con+SB

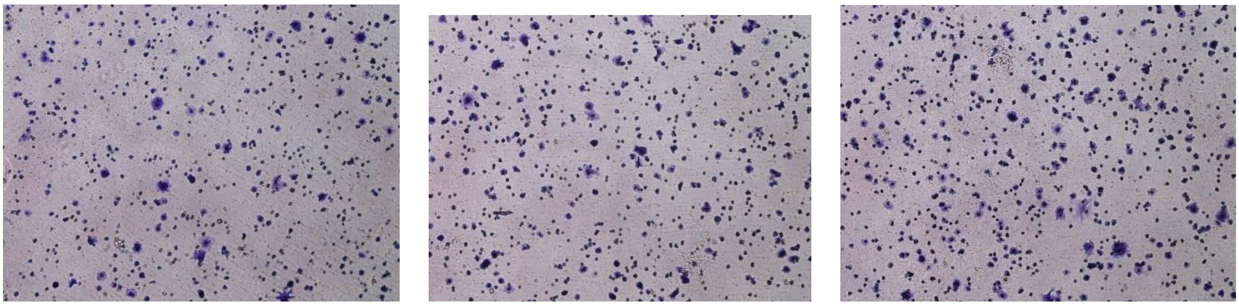

ADSC CM+SB

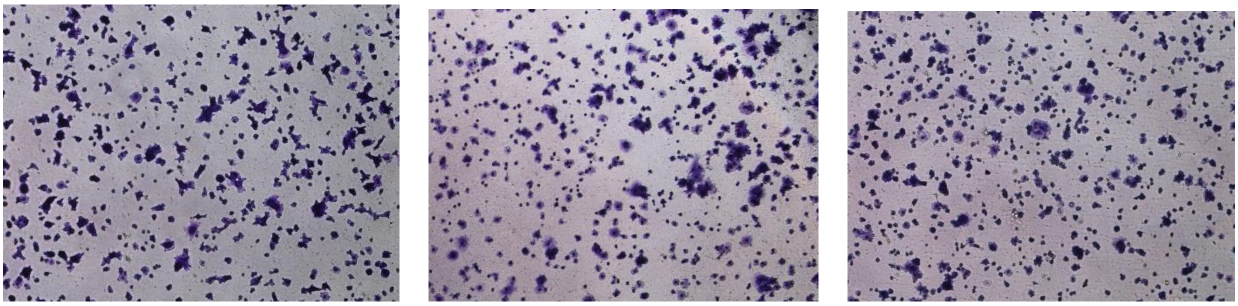

M Invasion (OV3)(CM+SB)

Con

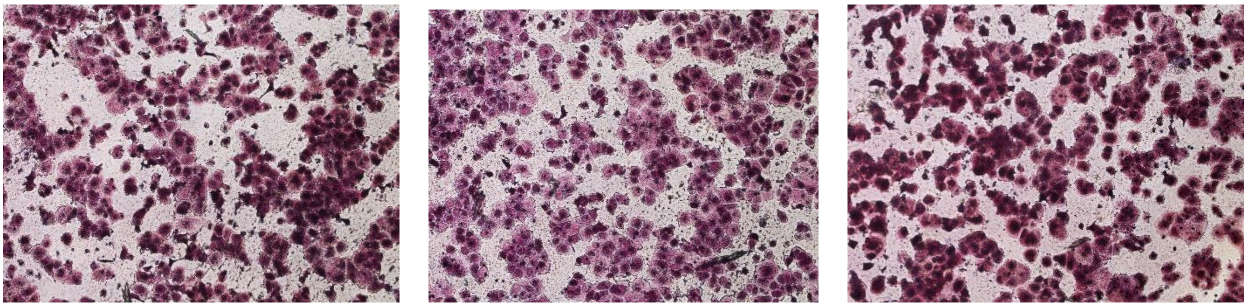

ADSC CM

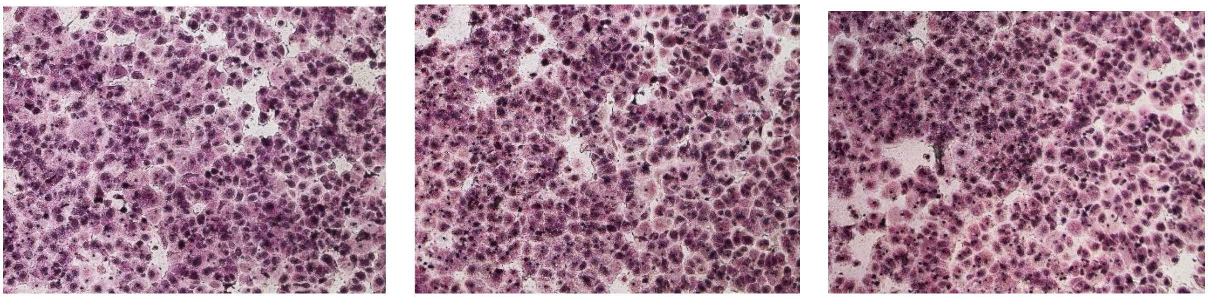

ADSC CM+SB

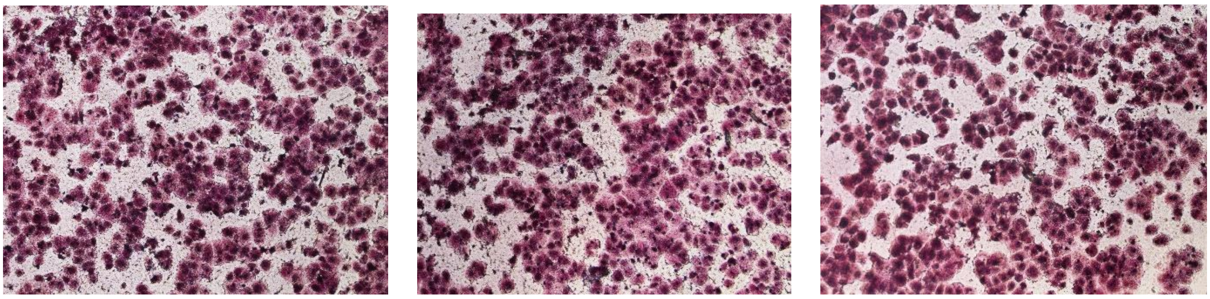

Con+SB

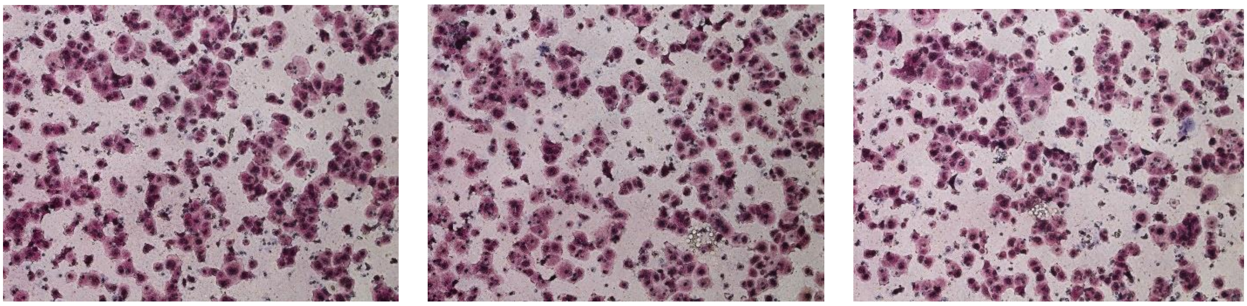

N Invasion (OV8)(CM+SB)

Con

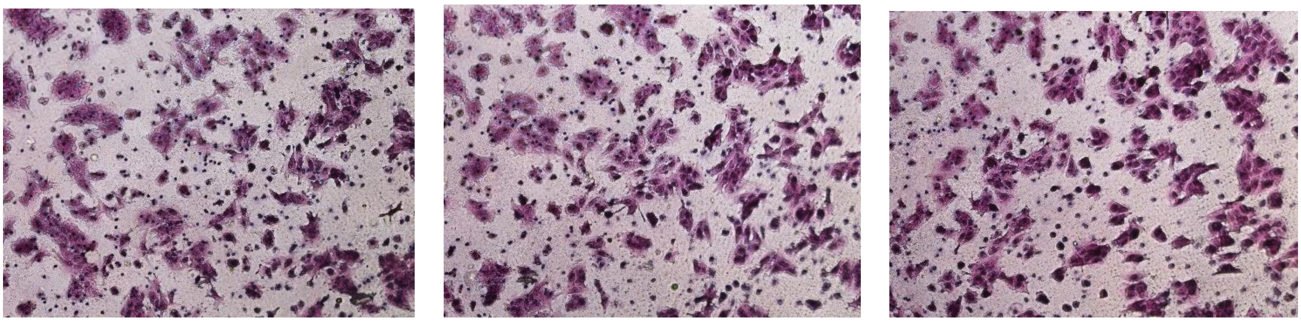

ADSC CM

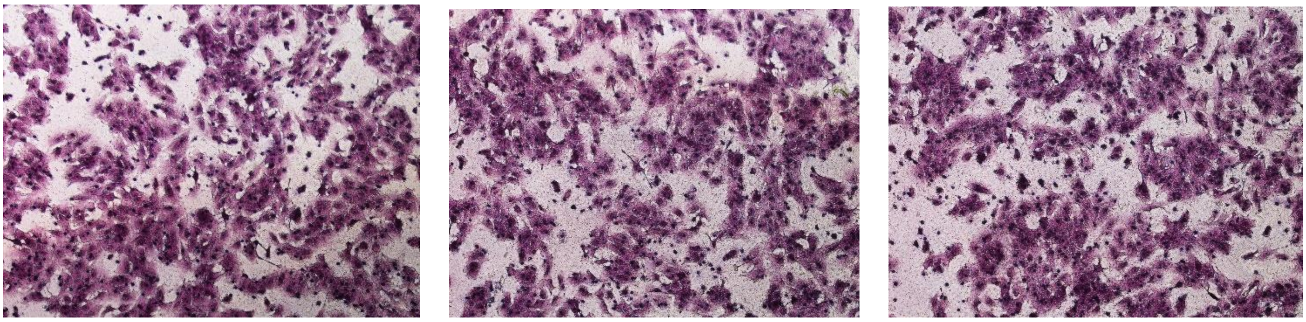

Con+SB

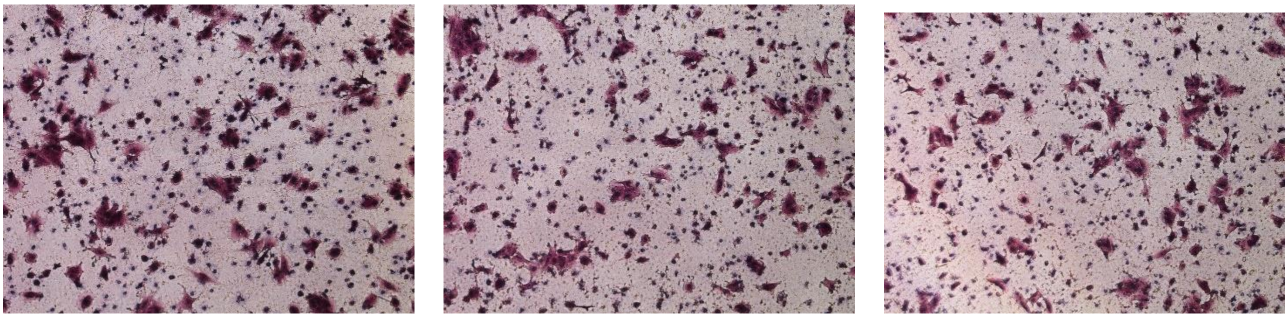

ADSC CM+SB

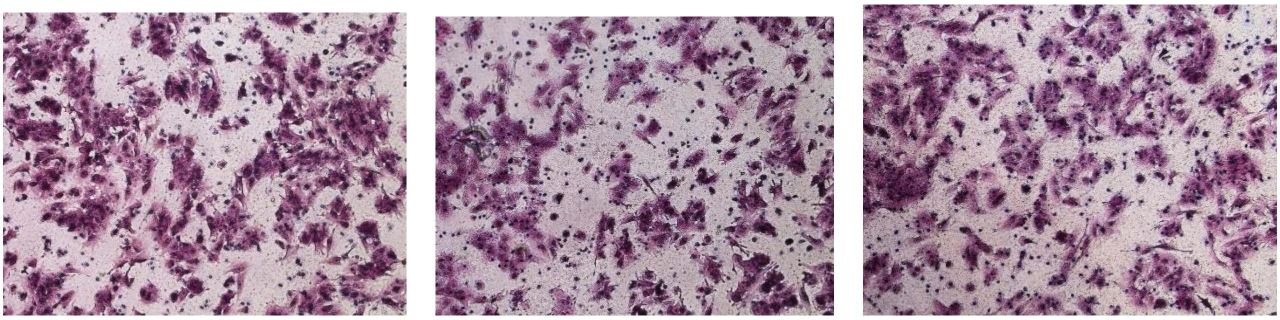

Figure 4

A Live animal imaging

Con

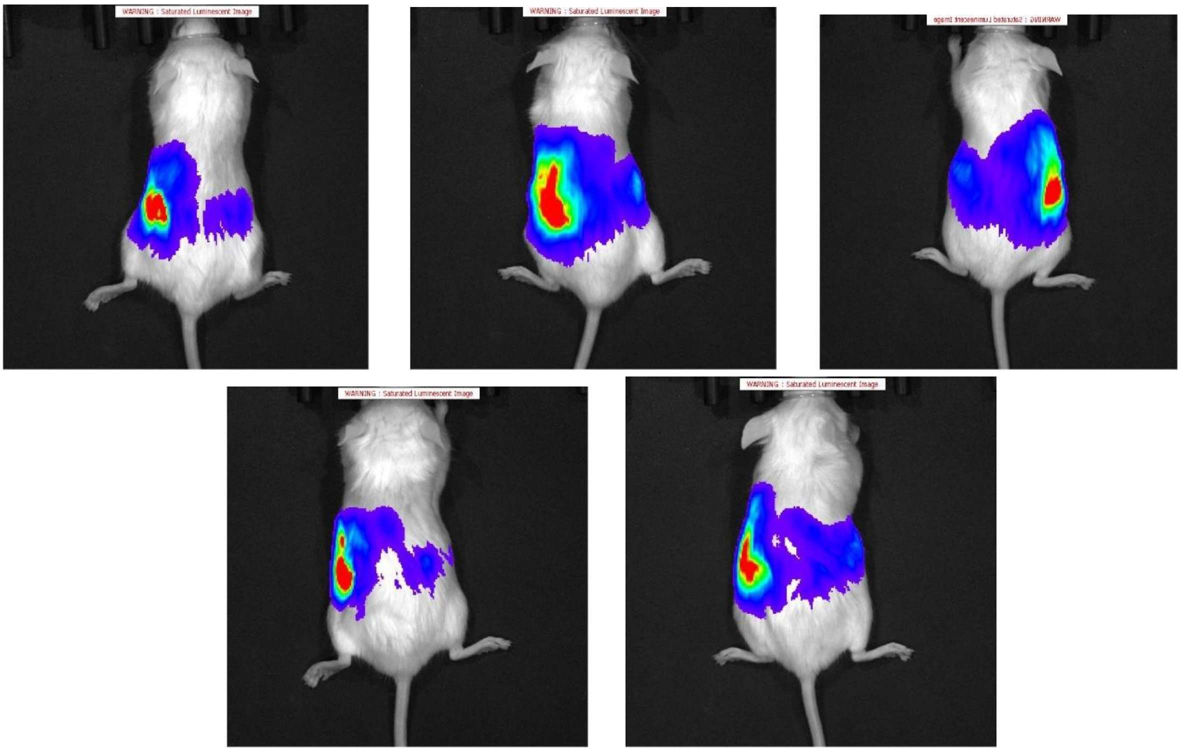

ADSC CM

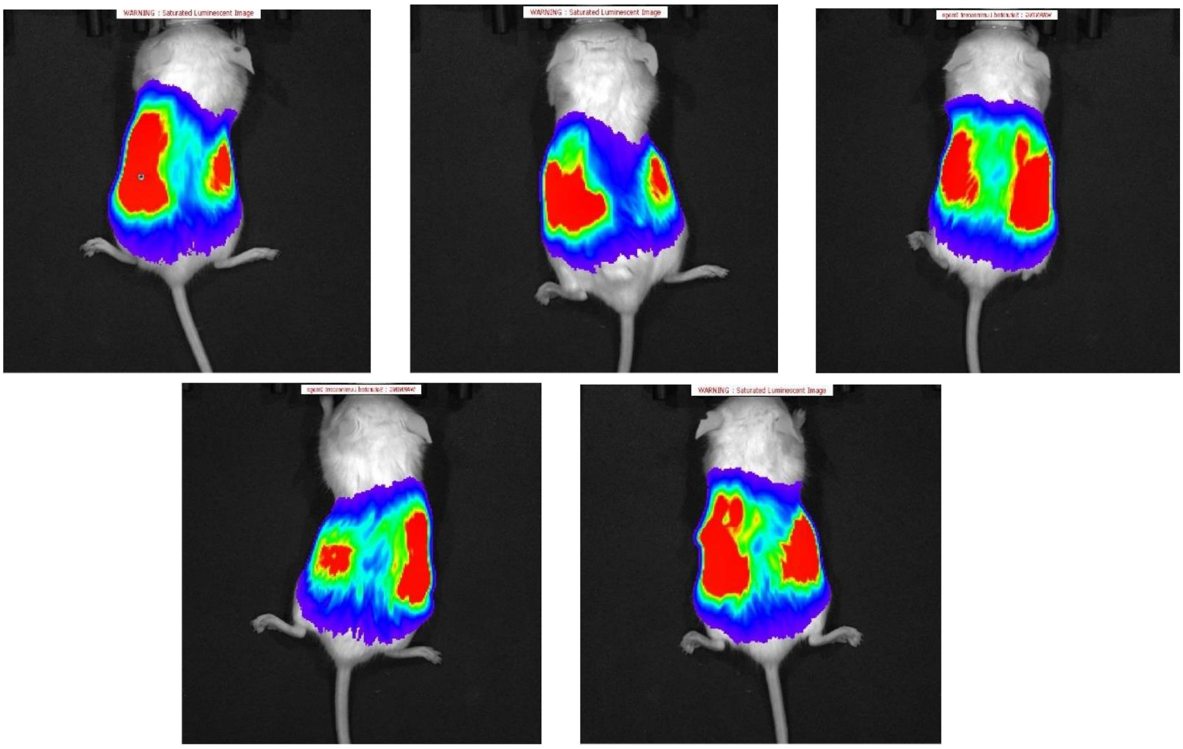

SB

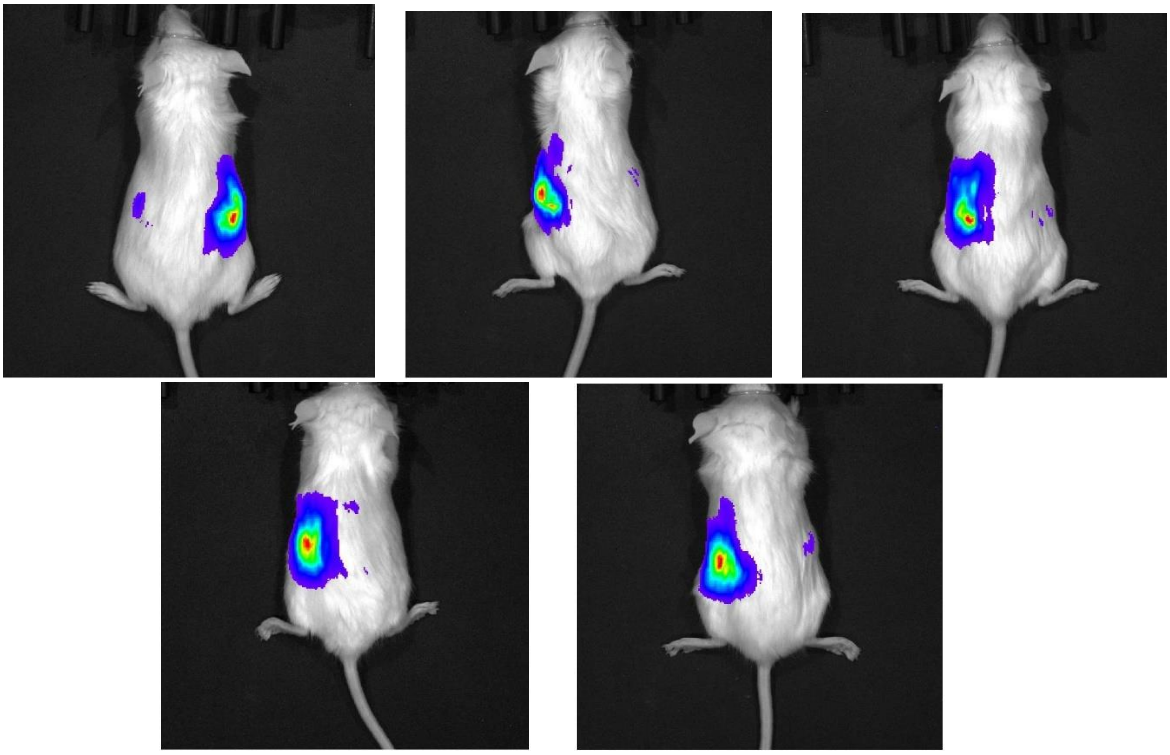

CM+SB

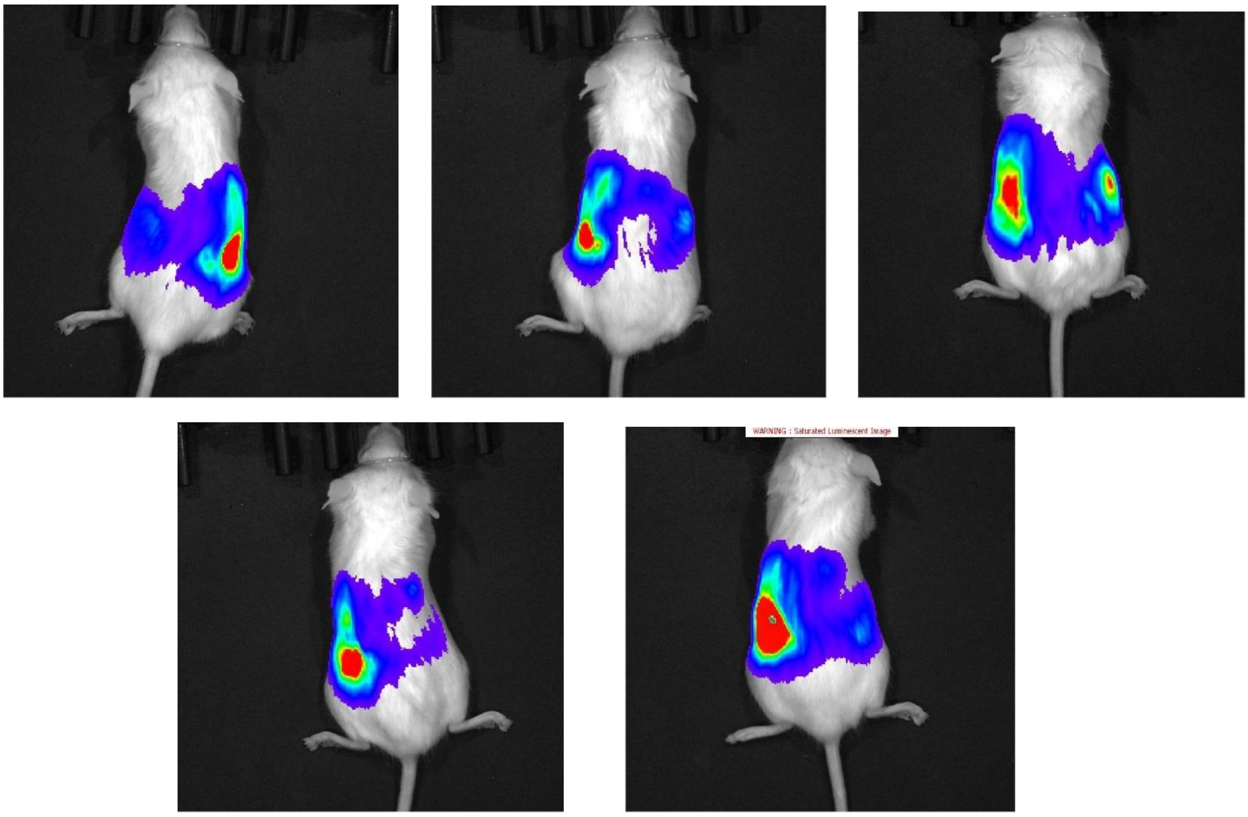

B H&E stained section of primary tumor

Con ADSC CM

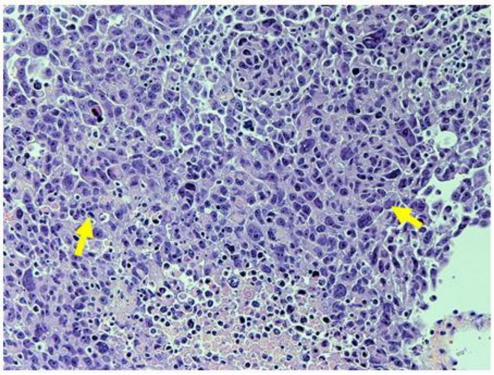

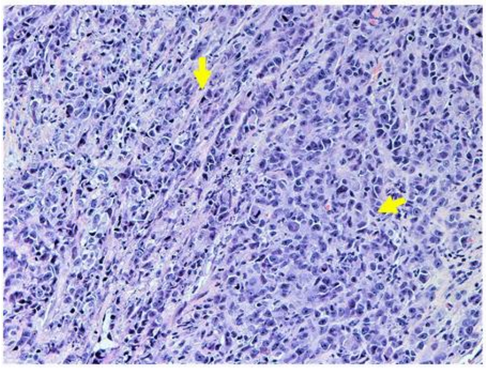

SB CM+SB

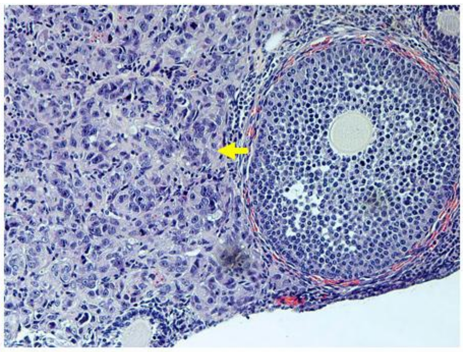

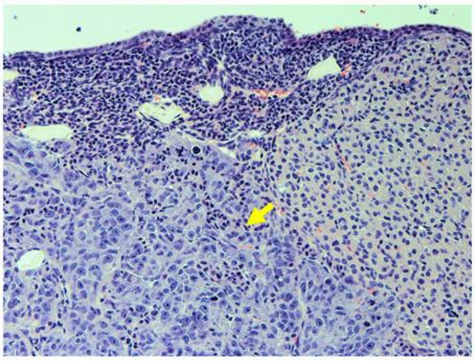

C primary tumors in ovaries by live animal imaging

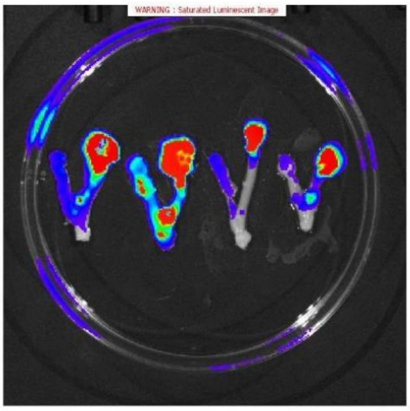

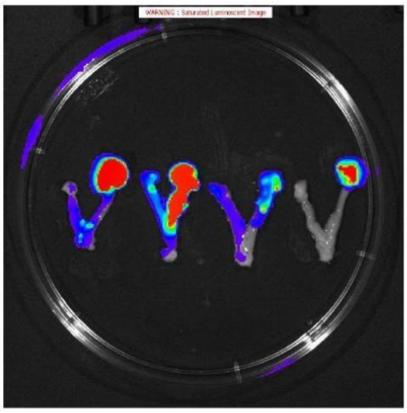

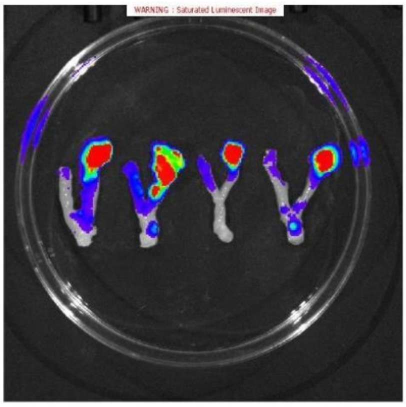

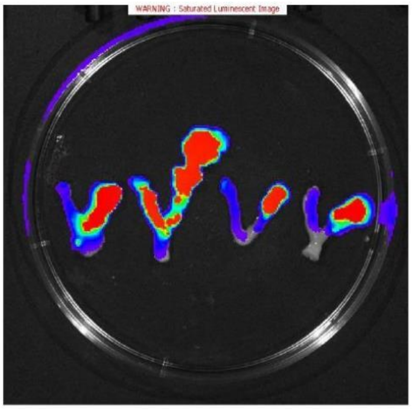

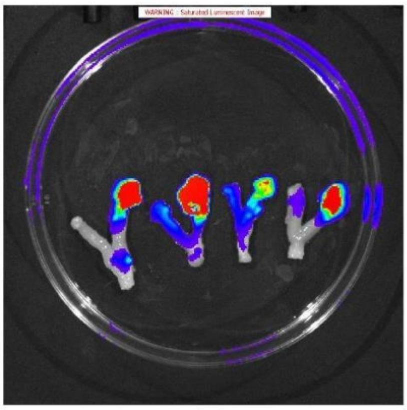

D tumors in ovaries of mice were imaged

Con ADSC CM SB CM+SB

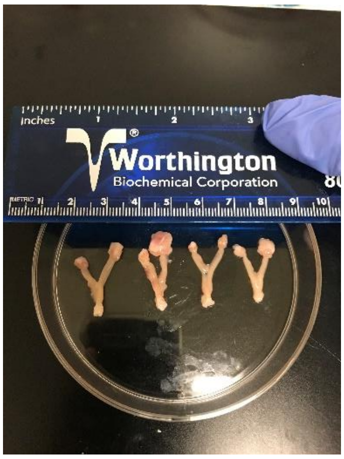

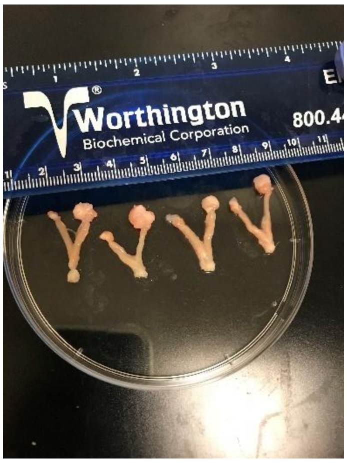

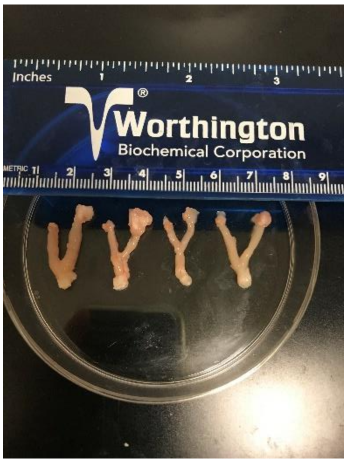

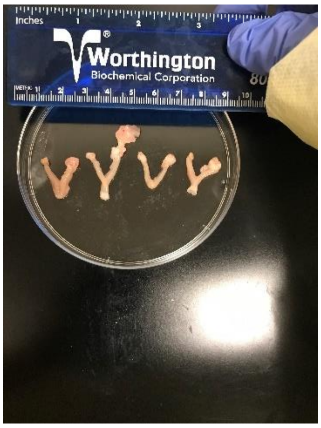

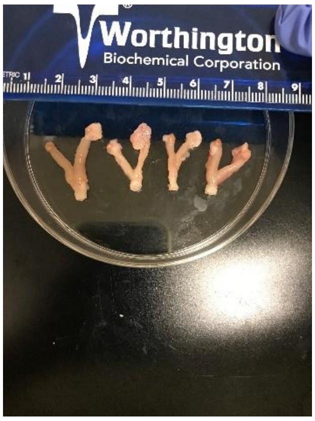

F western blot of p-SMAD2 and EMT markers in primary ovarian tumors

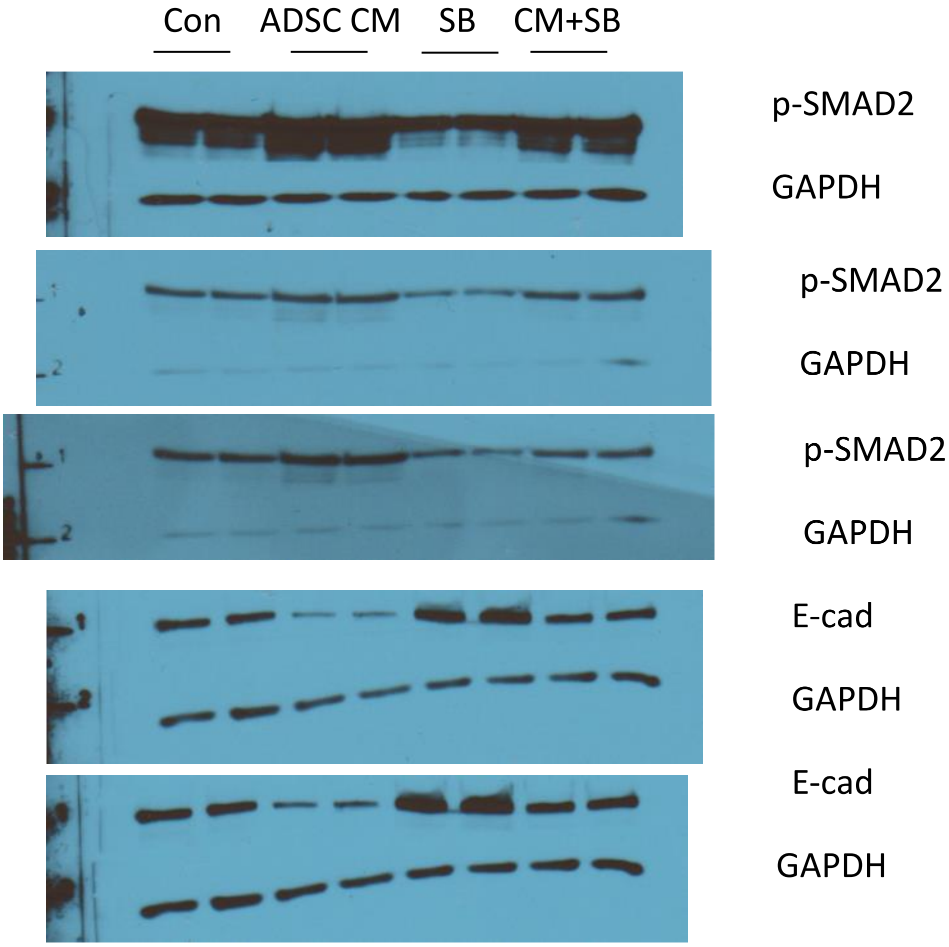

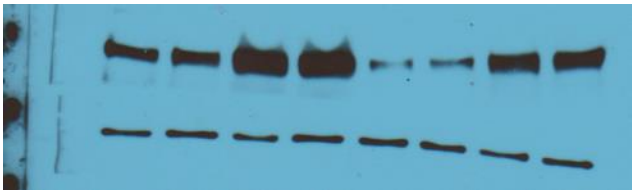

N-cad

GAPDH

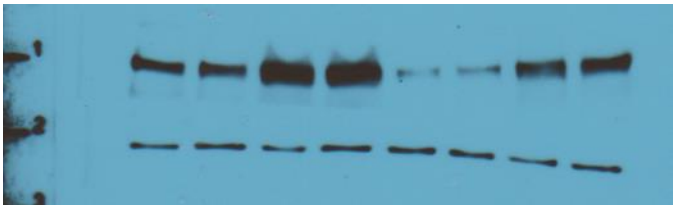

N-cad

GAPDH

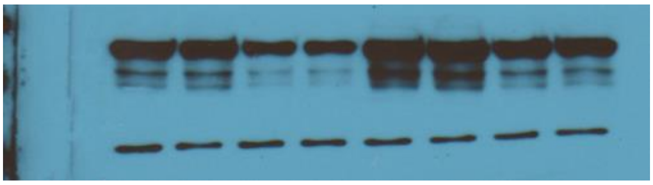

Cyt-7

GAPDH

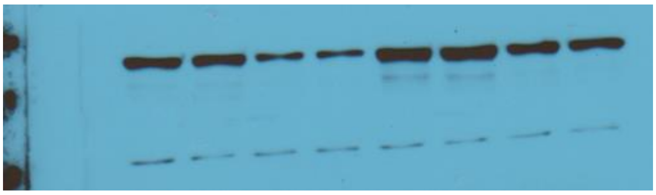

Cyt-7

GAPDH

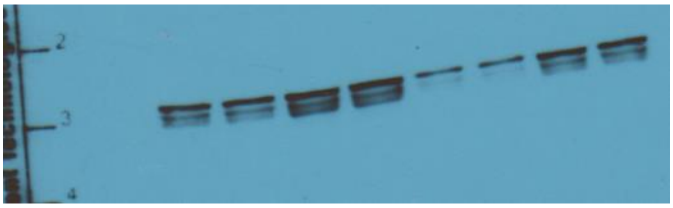

Vimentin

GAPDH

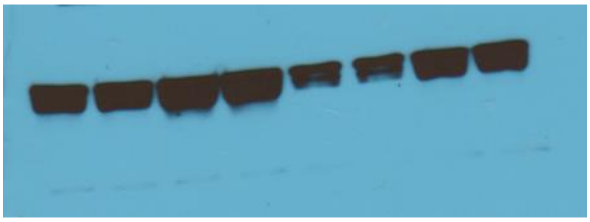

Vimentin

GAPDH

G metastatic tumors of xenografted mice by live animal imaging.

Liver

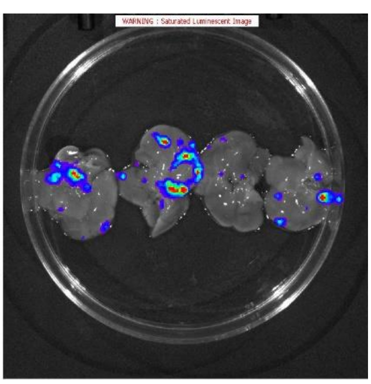 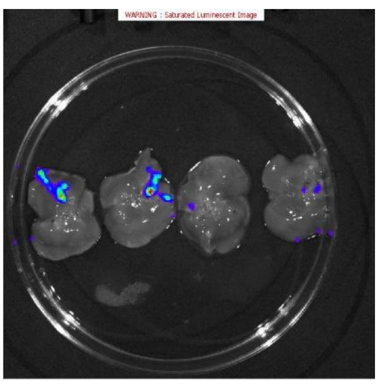 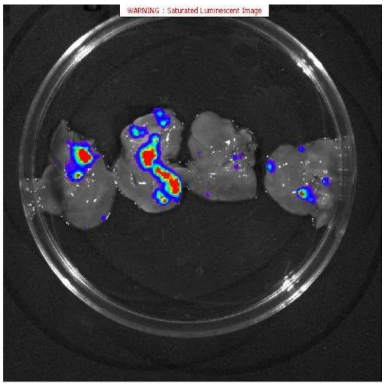

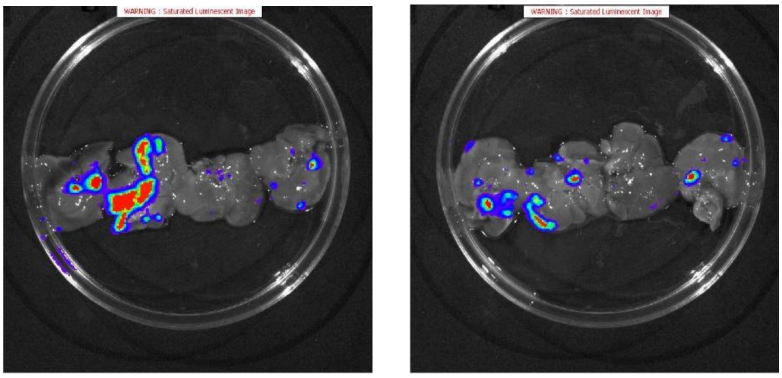

Spleen

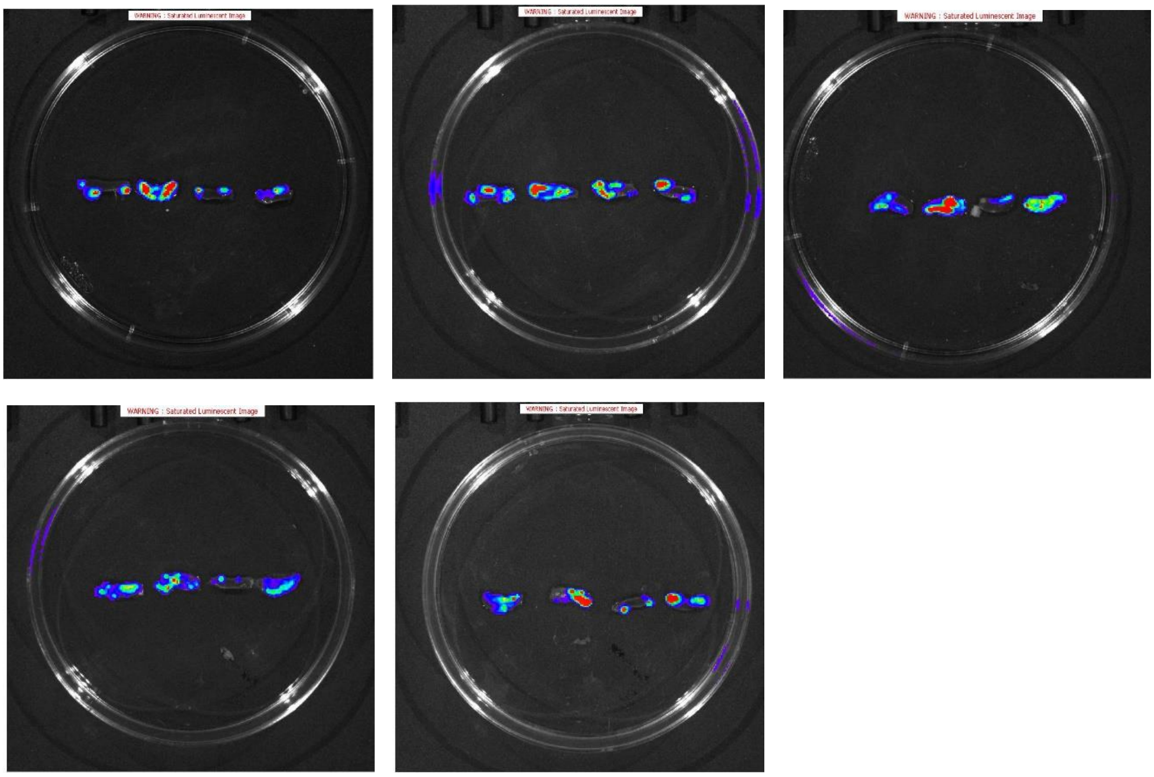

Kidney

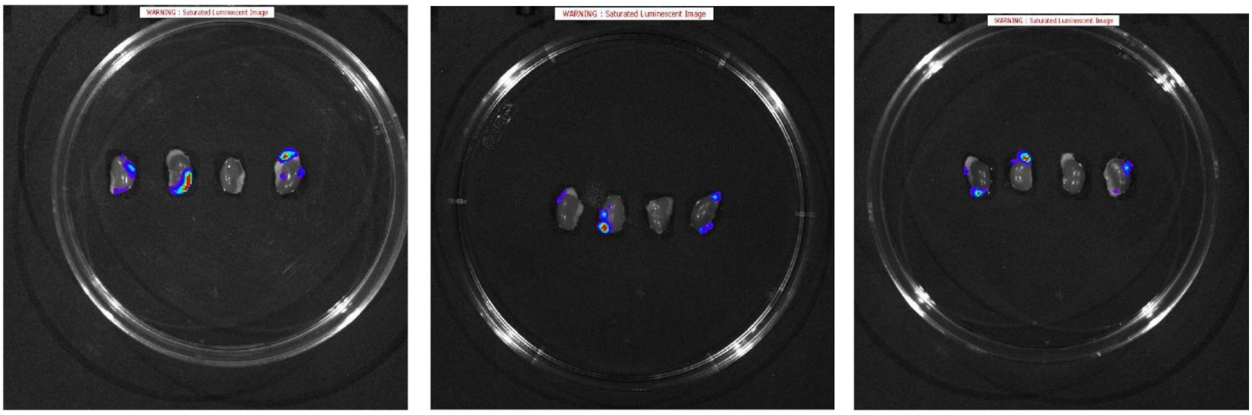

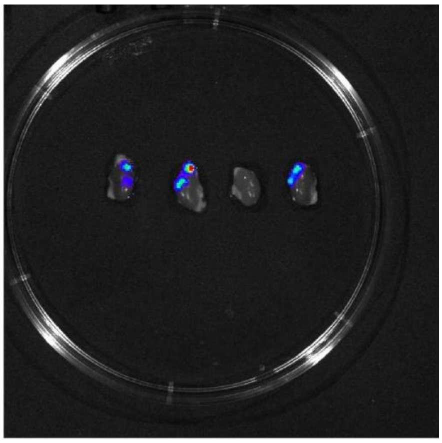

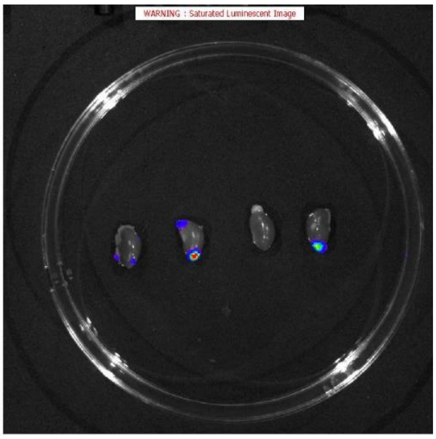

Intestine

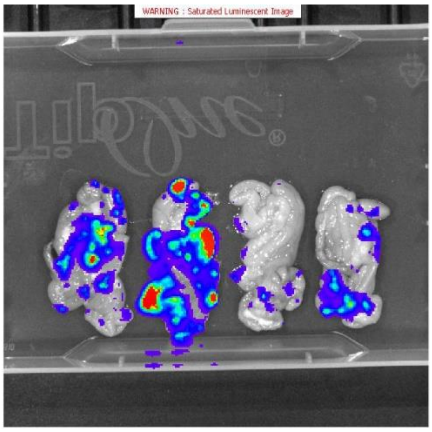

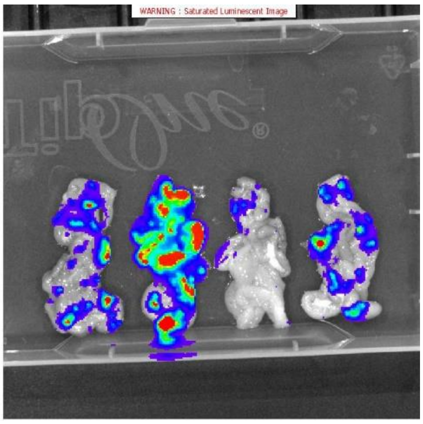

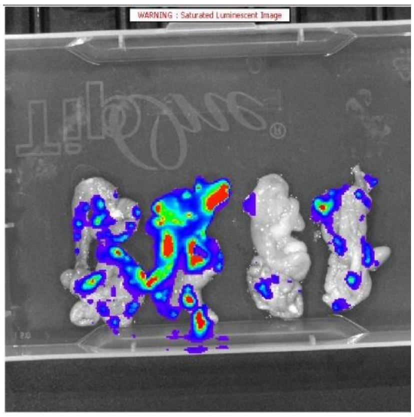

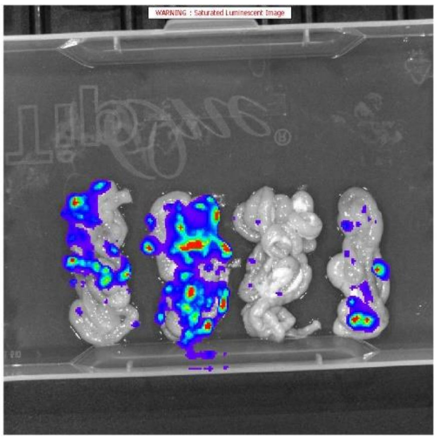

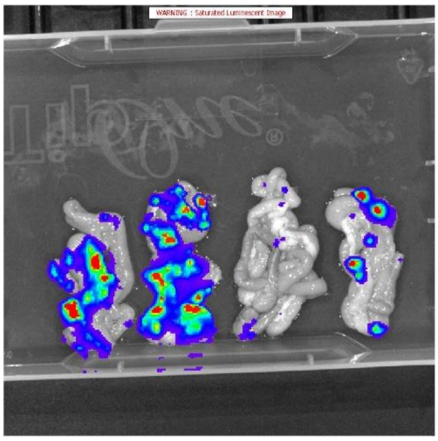

Stomach

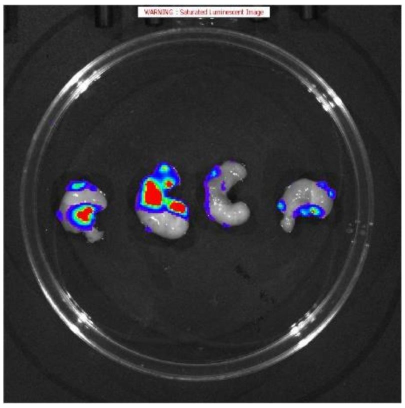

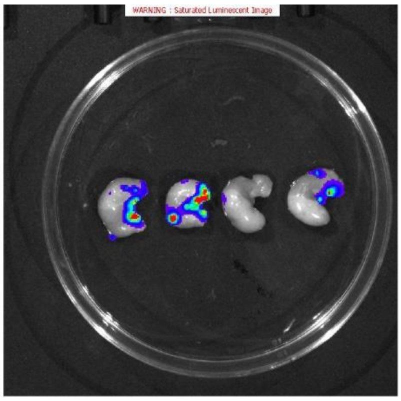

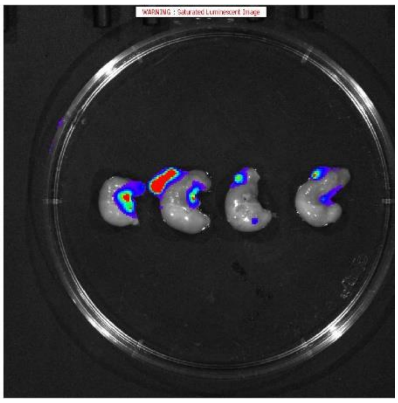

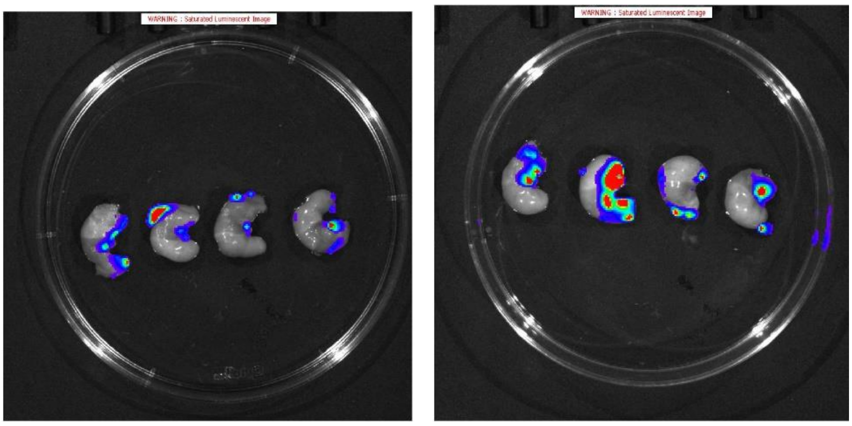

H Liver H&E staining of metastatic tumors

Con ADSC CM

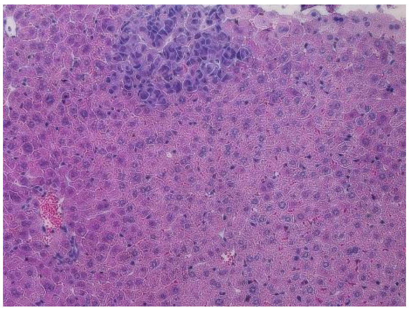

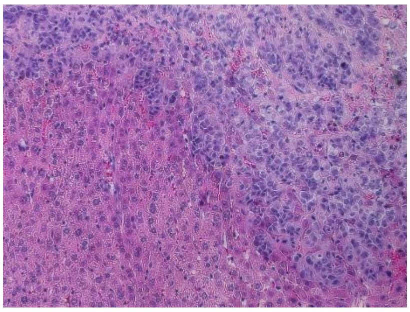

SB CM+SB

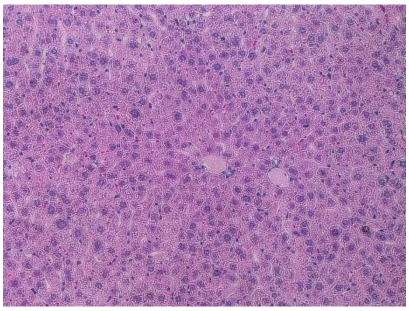

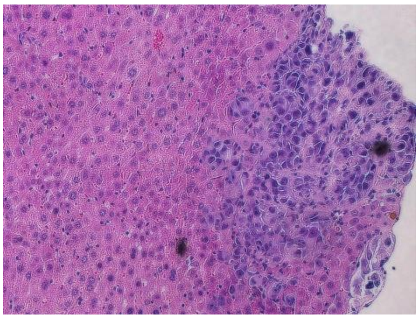

Supplement: Supplementary file 2 [file DataSheet_2.pdf]
